# Transcriptomic analysis reveals the gene regulatory networks involved in leaf and root response to osmotic stress in tomato

**DOI:** 10.3389/fpls.2023.1155797

**Published:** 2023-06-02

**Authors:** Raul Pirona, Giovanna Frugis, Franca Locatelli, Monica Mattana, Annamaria Genga, Elena Baldoni

**Affiliations:** ^1^ National Research Council (CNR), Institute of Agricultural Biology and Biotechnology (IBBA), Milano, Italy; ^2^ National Research Council (CNR), Institute of Agricultural Biology and Biotechnology (IBBA), Rome Unit, Roma, Italy

**Keywords:** *Solanum lycopersicum*, osmotic stress, transcriptomic data, leaf and root, gene co-expression network, transcription factors

## Abstract

**Introduction:**

Tomato (*Solanum lycopersicum* L.) is a major horticultural crop that is cultivated worldwide and is characteristic of the Mediterranean agricultural system. It represents a key component of the diet of billion people and an important source of vitamins and carotenoids. Tomato cultivation in open field often experiences drought episodes, leading to severe yield losses, since most modern cultivars are sensitive to water deficit. Water stress leads to changes in the expression of stress-responsive genes in different plant tissues, and transcriptomics can support the identification of genes and pathways regulating this response.

**Methods:**

Here, we performed a transcriptomic analysis of two tomato genotypes, M82 and Tondo, in response to a PEG-mediated osmotic treatment. The analysis was conducted separately on leaves and roots to characterize the specific response of these two organs.

**Results:**

A total of 6,267 differentially expressed transcripts related to stress response was detected. The construction of gene co-expression networks defined the molecular pathways of the common and specific responses of leaf and root. The common response was characterized by ABA-dependent and ABA-independent signaling pathways, and by the interconnection between ABA and JA signaling. The root-specific response concerned genes involved in cell wall metabolism and remodeling, whereas the leaf-specific response was principally related to leaf senescence and ethylene signaling. The transcription factors representing the hubs of these regulatory networks were identified. Some of them have not yet been characterized and can represent novel candidates for tolerance.

**Discussion:**

This work shed new light on the regulatory networks occurring in tomato leaf and root under osmotic stress and set the base for an in-depth characterization of novel stress-related genes that may represent potential candidates for improving tolerance to abiotic stress in tomato.

## 1 Introduction

Plants are continuously exposed to adverse environmental conditions, including water shortage, extreme temperatures, heavy metals, and salinity stress, that can seriously affect plant growth and development. Among abiotic stresses, drought showed the greatest impact on crop yield, especially in semi-arid and arid regions (Wada et al., 2011; Lesk et al., 2016). Additionally, climate changes are intensifying the frequency, duration, and severity of drought in further agro-environments. With the global temperature increasing and worldwide population growth, the scarcity of water resources in agriculture will aggravate crop loss (Wada et al., 2011; Trenberth et al., 2014). This projection is predicted to be severe in the Mediterranean region, including southern Spain and Italy, two major tomato producers (Saadi et al., 2015; Cammarano et al., 2020). Thus, the next generation of agriculture urgently needs new strategies to improve crop drought tolerance and water use efficiency and to develop more resilient crop varieties capable of surviving drought conditions while maintaining yield (Bailey-Serres et al., 2019).

Tomato (*Solanum lycopersicum* L.) is one of the major horticultural crops cultivated worldwide and is a key component of the diet for billion people. It also represents an important dietary source of vitamins A and C as well as carotenoids such as lycopene (Nasir et al., 2015). Most modern tomato cultivars are sensitive to water deficit, leading to a reduction in seed development and germination, impairing vegetative growth and reproduction (Saadi et al., 2015; Moles et al., 2018). Both vegetative and reproductive stages of modern tomato cultivars can be severely affected by drought, which inhibits seed development, reduces stem and fruit growth (Nuruddin et al., 2003). Despite this, some Mediterranean drought-tolerant tomato accessions show high intrinsic water use efficiency and advantageous physiological traits in the response to water deficit (Galmés et al., 2011; Galmés et al., 2013).

Plants have evolved several morphological, physiological, biochemical, and molecular mechanisms to overcome water stress (Fàbregas and Fernie, 2019). Water limiting conditions lead to changes in the expression of drought responsive genes in different plant tissues (Shinozaki and Yamaguchi-Shinozaki, 2007; Riemann et al., 2015). Transcription factors (TFs) are key players in the regulatory networks underlying plant responses to abiotic stresses (Golldack et al., 2014). Transcriptomic studies can significantly support the identification of genes and regulatory pathways underlying plant responses to environmental fluctuations, including drought (Morozova et al., 2009; Bashir et al., 2019; Baldoni, 2022). Networks have rapidly become an attractive approach to manage, display, and contextualize large “omics” data sets in order to obtain a system level and molecular understanding of biological key processes. Clustering gene expression data allows to identify substructures and groups of genes that may share a biological function or be under the same transcriptional control (Altman and Krzywinski, 2017). Gene Co-expression Networks (GCNs) are graphical representations of complex interactions where genes are represented by nodes, and edges connect genes that are significantly co-expressed or anti-correlated (Hu et al., 2016). Dense gene communities in such networks, commonly referred to as modules, often indicate that the member genes are functionally related (Liu et al., 2019). Recently, the traditional “global” gene co-expression networks are often replaced by more defined, context-specific and targeted ones. Such GCNs imply genetic correlations in specific biological contexts such as during development or in response to a stress (Gupta and Pereira, 2019). Integration of cluster analysis and context-specific GCN approaches has been proven to successfully identify transcriptional regulatory networks involved in a wide range of plant biological processes, including photosynthesis, development, and response to abiotic stress (Balaguer et al., 2017; Buti et al., 2019; Testone et al., 2019; Baldoni et al., 2021), and to increase prediction and ranking of the “hub” genes in a system in order to design targeted strategies for genetic improvement.

Regarding tomato, transcriptomic studies have been recently performed on different varieties to dissect the gene network involved in drought response. Considering leaf and root organs, most of these studies analyzed gene expression changes under drought in leaves (Gong et al., 2010; Iovieno et al., 2016; Mishra et al., 2016; Liu et al., 2017; Lee et al., 2018; Park et al., 2019; Zhou et al., 2019; Diouf et al., 2020; Bian et al., 2021), only few studies were focused on roots (Balestrini et al., 2019; Karanja et al., 2021). To our knowledge, only one paper analyzed the drought-related transcriptomic changes in both tomato leaves and roots (Zhang et al., 2019). Since many molecular factors may operate regardless of organ specificity, leaf and root organs should not be considered separately from the whole plant to understand the complex molecular network of drought response and its regulatory mechanisms. Finally, very few studies described GCNs involved in water stress response in tomato (Nicolas et al., 2022; Wei et al., 2023).

In the present work, a transcriptomic analysis in response to osmotic stress was conducted on two tomato genotypes, M82 and Tondo, an Italian cultivar with typical round-fruits used in the industrial tomato sector. The analysis was performed separately on leaves and roots to characterize the specific response of these two organs, and then the obtained data were integrated to elucidate the mechanisms involved in the osmotic stress response of the whole tomato plant.

## 2 Materials and methods

### 2.1 Plant material, growth conditions and treatments

For the osmotic stress treatment, seeds of M82, and of four Italian tomato genotypes (Tondino, Pisanello, Cuore di Bue, and Tondo) were used. The seeds of the four Italian varieties were kindly provided by CREA - Research Centre for Genomics and Bioinformatics in Montanaso Lombardo (Italy).

The seeds were sterilized with 70% ethanol for 5 min and subsequently with a solution of 10% NaOCl and 0.1% SDS for 10 min twice, thoroughly rinsed and then placed on dH_2_O-moistened Petri dishes at 26°C in the dark. After 5 days, the germinated seedlings were inserted into holes of black polystyrene foam sheets and transferred to plastic boxes containing the standard MS nutrient solution (Duchefa Biochemistry, Harlem, The Netherlands). To avoid hypoxia, continuous aeration of the medium was provided by an aquarium air pump *via* flexible plastic tubing. Nutrient solution was renewed every 2 days. To minimize any potential damage to the root system, plants were transferred into the new nutrient solution by moving the polystyrene foam sheets. All the hydroponic experiments were performed in a controlled growth chamber at 25°C/21°C under a 14 h light/10 h dark photoperiod with a light intensity of 200 µmol m^-2^ s^-1^ and 50% relative humidity. For the osmotic stress treatment, after 14 days of growth (third-leaf stage), half of the plants were transferred to a nutrient solution with 5, 10 or 20% polyethylene glycol (PEG) 6000 (Duchefa Biochemistry, Harlem, The Netherlands) at 11:00 am (3 hours after the light onset).

For RNA extraction, leaves and roots from control and treated plants were separately collected after 24 h (11:00 am) in three biological replicates, each consisting of six plants.

### 2.2 Physiological measurements

#### 2.2.1 Relative water content

The leaf relative water content (RWC) of untreated and 3 h or 24 h-treated plants was measured to determine the plant response to osmotic stress. The first and second leaves from each plant were collected, and the fresh weight (FW) was immediately measured. The leaf blades were then floated on deionized water for 24 h in the dark, and rehydrated leaves were reweighed to determine the turgid weight (TW). Finally, the leaf blades were oven dried at 70°C until constant weight, and the dry weight (DW) of each leaf was measured. The RWC was calculated using the following equation: RWC (%) = (FW – DW)/(TW – DW) x 100. For each genotype and condition, seven biological replicates were used.

#### 2.2.2 Electrolyte leakage assay

The leaf electrolyte leakage (EL) of untreated and 3 h or 24 h-treated plants was measured to evaluate cell membrane stability following the method by Shou et al. (2004) with minor modifications. For each sample, six leaves from three plants (two leaves per plant) were collected, rinsed with deionized water, and cut into discs of 7 mm diameter. Discs were incubated in tubes with 20 ml deionized water and the tubes were shaken over-night in a slanted position. The initial conductivity of the incubation (*C*i) was measured using a conductivity meter (Thermo Orion star Plus, Beverly, MA) to estimate the amount of ions released from cells under control conditions and PEG treatment. Leaf tissue in the incubation solution was then boiled at 100°C for 30 min to completely disrupt the cell structure. The conductivity of the boiled solution (*C*max) was determined after cooling at room temperature. These two measurements were carried out individually for all the samples from both control and stressed plants. The percentage of EL was calculated using the following equation: EL (%) = *C*i/*C*max x 100. For each genotype and condition, seven biological replicates were measured.

### 2.3 Total RNA extraction

Total RNA was extracted from leaves using the TRIzol^®^ RNA Purification Kit (Invitrogen, Carlsbad, CA) following the manufacturer’s instructions. RNA purity was checked spectrophotometrically (Nanodrop ND-1000 Spectrophotometer, Celbio, Italy) and only samples with absorbance reading ratio at 260 / 280 nm between 1.7 and 2.1 were used. The integrity of RNA was verified using the RNA 6000 Nano Labchip Kit on an Agilent 2100 Bioanalyzer (Agilent Technologies, USA) following the manufacturer’s protocol. Only samples with a 28S / 18S ratio ≥ 2 were used for further experiments.

### 2.4 Microarray hybridisation

The cDNA synthesis, labeling and hybridization were performed according to NimbleGen Arrays User’s Guide: Gene Expression Analysis (Version 5.1) by the Functional Genomic Center of the University of Verona (Italy; http://ddlab.sci.univr.it/FunctionalGenomics/). Each sample was hybridized to a custom NimbleGen microarray named 111012_Slyc_PT_expr (Roche, NimbleGen), which contains probes targeted to 28,191 tentative consensus sequences (TC; **Table S1**). Scanning was performed with an Axon GenePix 4400A scanner (Molecular Devices, San Jose, CA). Scanner settings were set according to NimbleGen gene expression user guide. Gene calls were generated using the Robust Multichip Average (RMA) algorithm as described by (Bolstad et al., 2003; Irizarry et al., 2003; Irizarry et al., 2012).

### 2.5 Conversion TC-Solyc

The 28,191 TC were blasted against the tomato reference cDNA database. The tomato reference multi-fasta file with cDNA sequences was downloaded from the Sol Genomics Network (https://solgenomics.net/) website as version ITAG4.0. Local blast alignment was performed using BLAST+ (version 2.10.1) with default parameters except for similarity that was set to ≥ 90%. A total of 28,097 TC blasted against the ITAG4.0 Solyc cDNA fasta file. Only 94 TC resulted in similarity below 90% or did not blast against any sequence. For multiple blast output, the best matching sequence was retained. As duplicated Solyc resulted, they were merged averaging the intensity values. After blast and merge, a total of 16,654 transcripts remained (**Table S1**).

### 2.6 Differential expression analysis

The differential expression analysis was conducted on both TC and Solyc raw intensity data. Raw data were log_2_ transformed and differential expression analysis was performed with LIMMA (Linear Models Microarray Analysis; Robinson et al., 2010; Ritchie et al., 2015) R package. *P*-values were adjusted for multiplicity with BH method (Benjamini and Hochberg, 1995). To identify differentially expressed transcripts (DETs), *P*-value was set to < 0.05 and the minimum fold change value was 2 (corresponding to values ≤ -1 and ≥ 1 in log_2_ scale (LFC); **Table S2**).

The TC differential expression analysis aimed to filter those TCs corresponding to the same Solyc but showing a contrasting differential expression (*i.e.*, log fold change (LFC) ≤ -1 in one TC and ≥ 1 in another TC corresponding to the same Solyc) between control and PEG. In such a case, when one TC showed an opposite value compared to the other TCs corresponding to the same Solyc, the Solyc was discarded. The Solyc differential expression analysis was used for further analyses. From now on, a transcript is defined as corresponding to a tomato reference transcript model as version ITAG4.0 (for example: Solyc06g084170.3.1).

### 2.7 GO-enrichment analyses

For each comparison, the DETs were submitted to the AgriGO v2.0 (http://systemsbiology.cau.edu.cn/agriGOv2/; Tian et al., 2017). To reduce the complexity of the GO terms, the Revigo webtool (http://revigo.irb.hr/) was used. From AgriGO v2.0 results, input data for Revigo analysis were GO IDs and the associated p-values. For tree map figures, the R scripts were downloaded from Revigo results and custom modified in R for better graphics layout (**Table S3**).

### 2.8 Annotation of transcription factors

In the microarray transcriptomic database, genes encoding TFs were annotated and classified by family based on TF annotations in the PlantTFDB database (Jin et al., 2014). TF annotation was enriched by integrating information from *Arabidopsis thaliana*, based on the best hit homology with *S. lycopersicum* deduced proteins.

### 2.9 K-means cluster analysis

The pipeline for the k-means cluster analysis was performed according to Testone et al. (2019) with some modifications. Briefly, means values of the Solyc raw intensity data, from three biological replicates of all DETs from both leaf and root samples (6,267 transcripts), were log-transformed using log2(x + 1) for data normalization. The optimum number of clusters was determined based on converging results of the sum of squared errors (SSE) estimate and the Calinsky criterion, as described by (Testone et al., 2019). Data scaling, k-means clustering, and visualization were performed in R according to the methods of Morgan et al. (2021). Cluster analysis data are available on **Table S4**.

### 2.10 Gene co-expression network

Considering the clusters selected for GCNs construction, only genes with a cluster score ≥ 0.8 were selected to perform pairwise correlation analysis of the expression values from the biological triplicates. We used the transcript raw intensity values of DETs in leaf and root from each biological replicate. Data from the different selected groups of clusters were log-transformed using log2(x + 1) for normalization, and Pearson pairwise correlation analysis was conducted across the selected samples using the “corrplot” and “hclust” packages of R software (Wei et al., 2017). Significant correlations (p-value ≤ 0.05), with a Pearson’s correlation coefficient (r) ≥ |0.9| were used to develop the GCNs. The Cytoscape software platform v. 3.5.1 (Shannon et al., 2003) was used to visualize the networks, to determine the relationships among the selected genes and to identify hub genes in the orchestration of the response to osmotic stress.

### 2.11 SWIM analysis

The SWItchMiner (SWIM) software (Paci et al., 2017) was used to predict key regulatory genes (“*switch genes*”) of PEG-induced stress response in tomato leaf and root. Raw intensity data from either leaves or roots of M82 and Tondo genotypes, in control or PEG-treated samples, were used to create the objects “list” and “matrix” and feed the software. The SWIM parameters used were: log2 transformation = yes; maximum percentage of allowed zeros and the minimum percentile for the IQR = 75% and 11th percentile default values, respectively; thresholds for the linear fold-change and for the FDR = 2 and 0.05 default values, respectively; threshold for the Pearson correlation coefficient = 85, corresponding to a correlation threshold of 0.98; maximum number of iterations allowed for each replicate of k-means, number of replicates for a given number of clusters, and maximum number of clusters for the scree plot = 100, 5, and 10 default values, respectively; chosen number of clusters = 3. The results of SWIM analysis are shown in **Table S5**.

### 2.12 Quantitative real-time PCR

The cDNA was synthesized using 0.5 μg of total DNase I-treated RNA using the SuperScript III First-Strand Synthesis SuperMiX for qRT-PCR, according to the manufacturer’s instructions (Invitrogen, Carlsbad, CA). qRT-PCR was performed using 20 µl triplicate reactions on a 7300 Real-Time PCR System (Applied Biosystems, USA) containing 5 µl of 1:10 diluted cDNA, a 300 nM final concentration of each primer and 10 μl of SYBR Green PCR Master Mix (Applied Biosystems, USA). The cycling program was as follows: 50°C for 2 min (1 cycle), 95°C for 10 min (1 cycle), 95°C for 30 s and 60°C for 1 min (40 cycles). The primer sets were tested by dissociation curve analyses and verified for the absence of nonspecific amplification. The dissociation curves were constructed using the following conditions: denaturation at 95°C for 15 s, cooling to 60°C for 30 s and then gradual heating at 0.01°C s^-1^ to a final temperature of 95°C. For each primer pair, calibration curves were generated with different dilutions and were accepted when the correlation coefficient was ≥ 0.99 and the efficiency was 1 (± 0.1). The relative expression levels were calculated using the 2^-ΔΔCt^ method (Livak and Schmittgen, 2001). Negative controls without cDNA were routinely included. The sequences of the primers used for qRT-PCR are shown in **Table S6**. The *UBI* gene (accession number TC193502) served as an endogenous reference using primers reported by Løvdal and Lillo (2009).

## 3 Results

### 3.1 Selection of tomato genotypes and experimental conditions for the osmotic stress treatment

The present work aimed to characterize the transcriptomic response to osmotic stress occurring in both leaves and roots of tomato. To mimic a stress imposed by a drying soil, we selected PEG 6000 that causes osmotic stress. We performed preliminary experiments to define a stressful condition for tomato plants at vegetative stage. In addition to the genotype M82, we used four Italian cultivars for the preliminary experiments: Tondino, Pisanello, Cuore di Bue, and Tondo. Among them, Pisanello is known to be drought sensitive, whereas Cuore di Bue shows an intermediate phenotype (Conti et al., 2019; Conti et al., 2021). For Tondo and Tondino, no previous data were available.

We tested two time points (3 and 24 h) and three PEG concentrations (10, 15 and 20%), according to literature (Li et al., 2022; Zhao et al., 2023), and measured leaf RWC and EL to evaluate stress response of tomato plants (**Table S7**). Only the treatment with 20% PEG concentration for 24 h resulted in both RWC and EL values being significantly different between control and PEG-treated plants for most of the genotypes, whereas no differences, or only differences in EL values, were observed at PEG 10% or PEG 15%, respectively. Hence, we opted for an incubation time of 24 h with PEG 20% to assure all genotypes sensed the osmotic stress. Tukey test conducted on both RWC and EL data from all the genotypes showed that Tondo and Cuore di Bue were significantly different compared to M82, whereas Tondino and Pisanello resulted similar to M82 (**Table S7**). We then selected M82 and Tondo genotypes for the transcriptomic analysis of this study.

### 3.2 Differential gene expression data in response to osmotic stress

**Figure 1:**
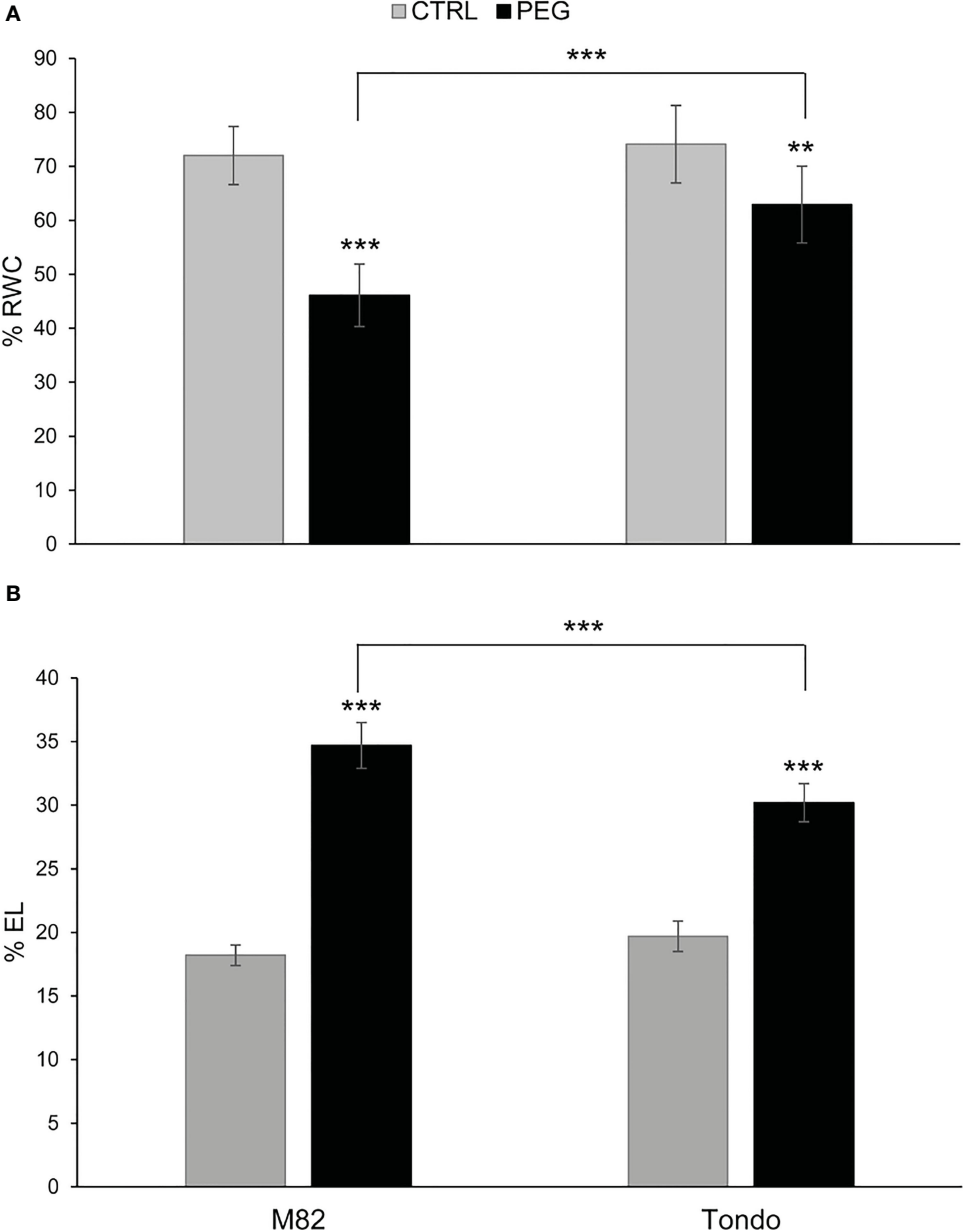
Physiological measurements of tomato plants in osmotic stress experiment: **(A)** Relative water content (RWC) and **(B)** electrolyte leakage (EL) measurements. Each percentage value is the mean ± SD of seven samples. Comparisons of differences were performed with Student’s *t*-tests (**: *P* ≤ 0.005; ***: *P* ≤ 0.001). The asterisks on the black bars represent the differences between control and PEG-treated plants within the same genotype. The asterisks above the brackets represent the differences between PEG-treated plants of the two genotypes.

M82 and Tondo were subjected to a 20% PEG treatment for 24 h and both RWC and EL data were measured. A reduction of RWC (46.1% in M82 and 62.9% in Tondo) and an increase of leaf EL (34.7% in M82 and 30.2% in Tondo) was observed in PEG-treated compared to control plants, (**Figure 1**), confirming that M82 was slightly but significantly more sensitive than Tondo to osmotic stress.

To investigate the molecular basis of the response to osmotic stress, a transcriptome analysis was conducted. The RNA samples were isolated from leaves and roots of control plants and 24 h PEG-treated plants. Three biological replicates were used for each genotype and condition, for a total of 24 samples. The 24 RNA samples were subjected to microarray hybridization and analysis, as described in Materials and Methods. The Principal Component Analysis (PCA) indicated the grouping of the biological triplicates, that clustered together. The samples clustered by treatment in the first PC (40.1% of the variance explained) and by the tissues type in the second PC (37.9% of the variance explained; **Figure S1**).

**Figure 2:**
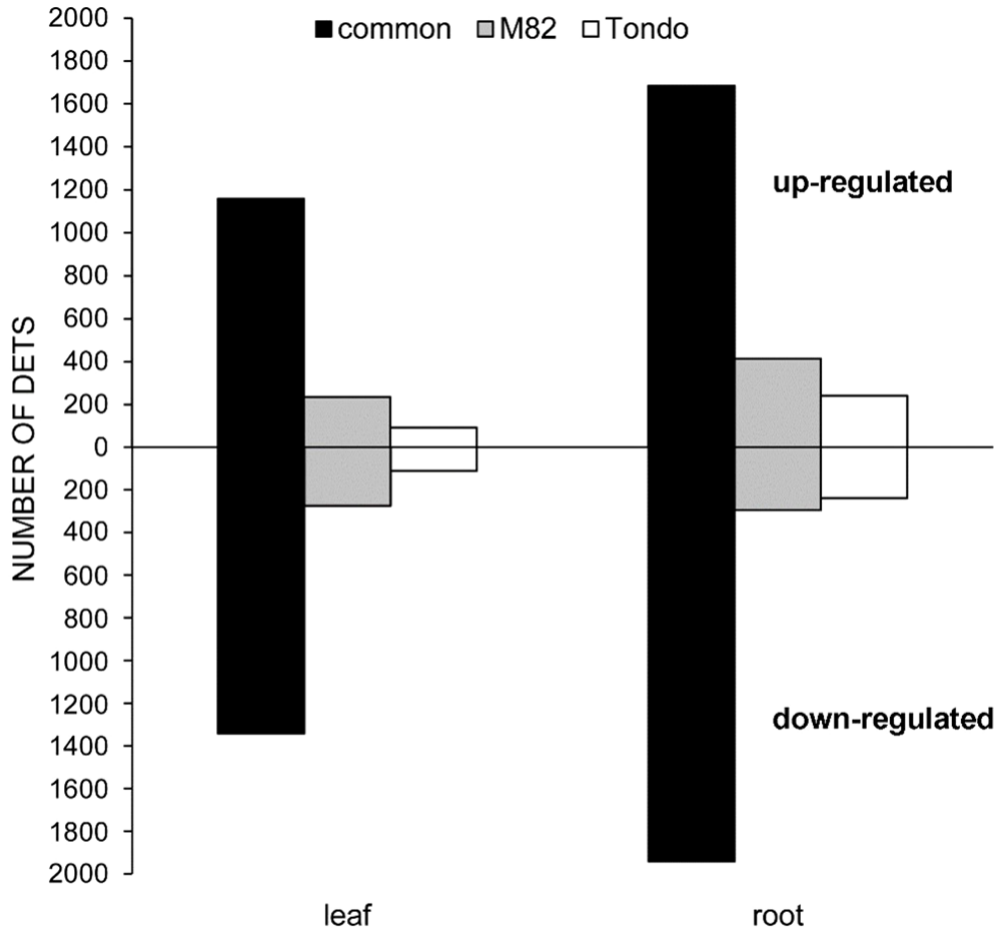
Bar chart of up- and down-regulated genes in leaf or root at 24 h of PEG treatment. Black bars: common regulated genes in the two genotypes. Grey bars: M82 specific up- or down-regulated genes. White bars: Tondo specific up- or down-regulated genes.

Osmotic stress caused significant changes in gene expression in leaf and root of both genotypes (**Table S2**). A total of 6,267 differentially expressed transcripts (DETs) were detected, of which 5,845 DETs in M82 and 5,417 in Tondo (**Table S2**). In both genotypes, more DETs were found in root compared to leaf, and the number of down-regulated transcripts resulted slightly higher than the number of up-regulated transcripts (**Table 1** and **Figure 2**). The stress response of the two genotypes consisted of DETs common to both genotypes as well as genotype-specific DETs, with the common response highly prominent in both leaves and roots (**Table 1** and **Figure 2**). Changes in gene expression of selected genes, identified as DETs in the microarray experiment, were validated using qRT-PCR. The comparison between microarray and qRT-PCR fold change data revealed a significant agreement supporting us for subsequent analyses (**Figure S2** and **Table S8**).

**Table 1:** Summary of the total number of DETs, DETs common to both cultivars and cultivar-specific DETs.

Organ	Total DETs		Common DETs	Specific DETs	
	M82	Tondo		M82	Tondo
leaf	3,010	2,705	2,501	509	204
root	4,333	4,107	3,627	706	480

**Figure 3:**
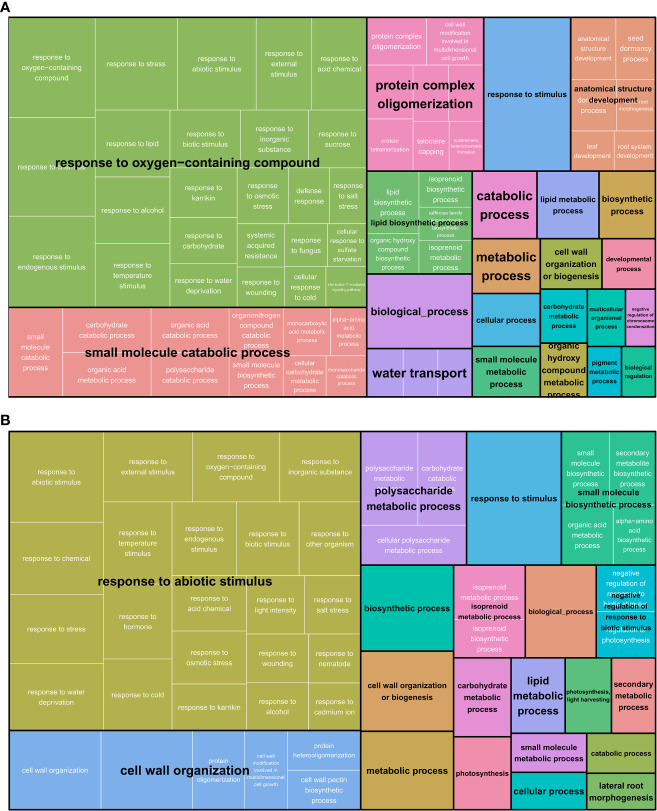
Revigo Tree Map of Biological Process in both M82 and Tondo genotypes in leaf **(A)** and root **(B)**. White text represents the original GO categories. Black text is the representative term of the redundant GO categories.

To inspect differences in GO terms and reveal biological trends in response to osmotic stress, AgriGO and Revigo analyses were performed separately on leaf and root samples, merging the DETs of the two genotypes (**Figure 3** and **Table S3**). In both tissues, a response to stress/stimulus (“response to oxygen-containing compound” in leaf and “response to abiotic stimulus” in root) was present and was the most abundant category. Furthermore, in leaf, “small molecule catabolic process” and “protein complex oligomerization” were the main categories represented. In roots, “cell wall organization” was highly represented followed by “polysaccharide metabolic process”.

**Figure 4:**
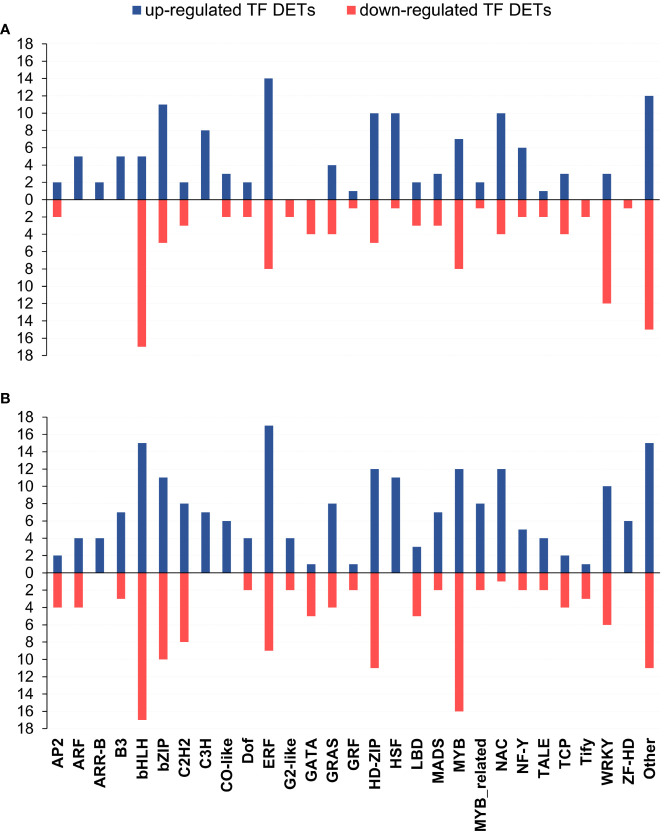
Bar chart showing per-class distribution of up- and down-regulated TF-encoding DETs in leaf **(A)** and root **(B)**. X axis indicates the TF class, Y axis indicates the number of TF-encoding DETs in the specific class.

Among the 6,267 DETs, we identified 439 TF-encoding genes: 246 and 341 in leaves and roots, respectively (**Table S9** and **Figure S3**). The most represented TF classes were bHLH, ERF, HD-ZIP and MYB (**Table S9** and **Figure 4**). The TF classes were differently distributed in leaves and roots as reported in **Figure 4**. In leaves, some classes (ARF, ARR-B, B3, and C3H) resulted to be exclusively up-regulated, whereas the classes G2-like, GATA, Tify and ZF-HD were exclusively down-regulated. In roots, ARR-B and C3H, CO-like, HSF and ZF-HD were exclusively up-regulated, while no TF classes showed an exclusive down-regulation.

### 3.3 Comparison between osmotic stress experiment and literature data on water stress at vegetative stage in tomato

To investigate possible similarities in the transcriptomic response to the osmotic treatment and to drought stress, we compared our dataset with previous gene expression analyses related to water stress in tomato.

We searched for specific genes that have been reported as stress markers in tomato. For leaf, among the 220 drought-related DEGs reported by Zhou et al. (2019), 103 resulted DETs in our leaf samples. For root, 10 genes out of 14 marker genes for water stress reported by Ferrández-Ayela et al. (2016) have also been detected in our dataset.

A recent study analyzed the transcriptomic response to drought stress in leaf at seedling stage of M82 and IL-9, a drought-tolerant line (Liu et al., 2017). The authors detected 2,828 stress-related DEGs for M82. Among them, 1,167 genes were differentially regulated also in our M82 leaf samples. Another study reported a transcriptomic analysis of drought stress in M82 plants at vegetative stage (Iovieno et al., 2016). They considered two cycles of drought stress (Dr1 and Dr2) and rewatering (RW) in comparison with watered plants (WW) and found 119 DEGs common to all four comparisons. Among these genes, 96 resulted as DETs also in our M82 leaf samples. These data gave reliable indications that the transcriptional changes observed upon PEG-mediated osmotic stress is in part overlapping with a medium/severe drought stress and can be useful to discover gene expression changes related to water stress response.

### 3.4 Clustering analysis

**Figure 5:**
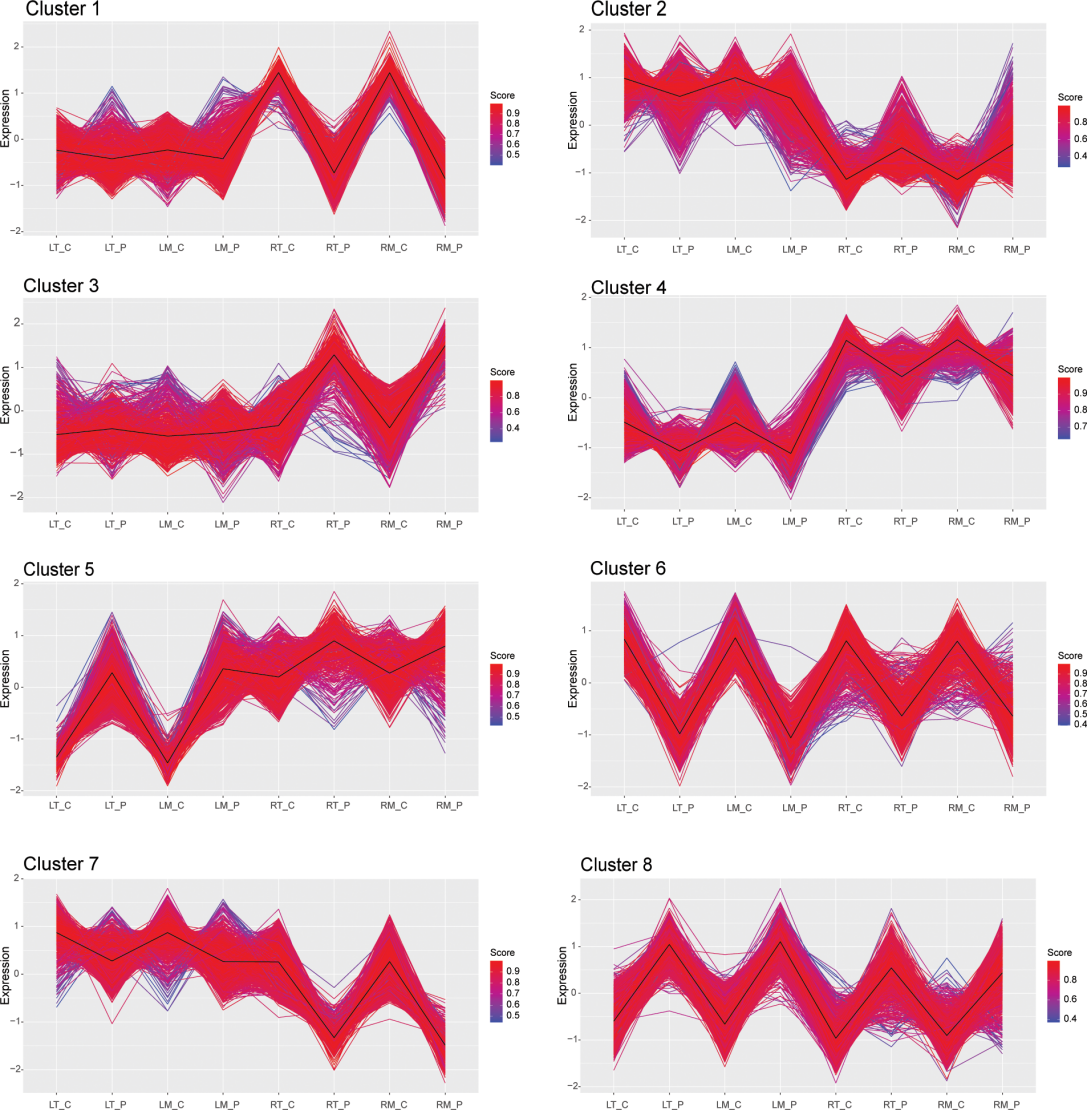
K‐means cluster analysis of DETs in the leaves and the roots of M82 and Tondo samples in control and 24 h-osmotic treatment. Distance matrix for k‐mean clustering was calculated by Euclidean similarity measurement and using centered Pearson’s correlation as the distance metric, resulting in eight gene clusters. Genes with a profile close to the core have a score approaching one (red) while those with divergent patterns have a score closer to zero (blue). Thick lines show centroid tendencies in each cluster. LT_C: Tondo leaves, control. LT_P: Tondo leaves, PEG-treated. LM_C: M82 leaves, control. LM_P: M82 leaves, PEG-treated. RT_C: Tondo roots, control. RT_P: Tondo roots, PEG-treated. RM_C: M82 roots, control; RM_P: M82 roots, PEG-treated.

To identify groups of genes with similar expression profiles in response to osmotic stress, we performed a k-means cluster analysis using the gene expression values of all the DETs identified in both leaves and roots of M82 and Tondo. This analysis defined the presence of eight different clusters of gene expression (**Figure 5** and **Table S4**). Amongst the eight clusters, clusters 6 and 8 contained DETs that were strongly down and up-regulated, respectively, in response to stress, in both leaves and roots. Cluster 4 contained genes that showed different transcript levels under control conditions in the two plant organs (higher in roots than in leaves) but with a slight down-regulation in both leaves and roots in response to the treatment. Revigo analysis of these three clusters showed categories related to response to stress, secondary metabolism, protein hetero-oligomerization, negative regulation of chromatin condensation, DNA replication (**Table S10**). Cluster 5 was characterized by a strong up-regulation in leaves and a slight up-regulation in roots in response to the treatment, and the main Revigo categories were related to stress response and small molecule metabolism (**Table S10**). Clusters 1 and 3 genes were strongly down and up-regulated in roots in response to stress, respectively. Cluster 7 was characterized by a strong down-regulation in roots in response to treatment and a minor effect in leaves. Revigo analysis of clusters 1, 3 and 7 showed ribosome biogenesis, rRNA metabolism, response to stimulus, polysaccharide metabolism, cell wall, root development, and small molecule biosynthetic processes as the main categories in these three clusters (**Table S10**). Finally, cluster 2 contained DETs that showed a stress-mediated down-regulation in leaves and a stress-mediated up-regulation in roots. This cluster was enriched in genes involved in photosynthesis, as shown by the Revigo analysis (**Table S10**).

### 3.5 Characterization of the pathways involved in the osmotic stress response in tomato leaves and roots

**Figure 6:**
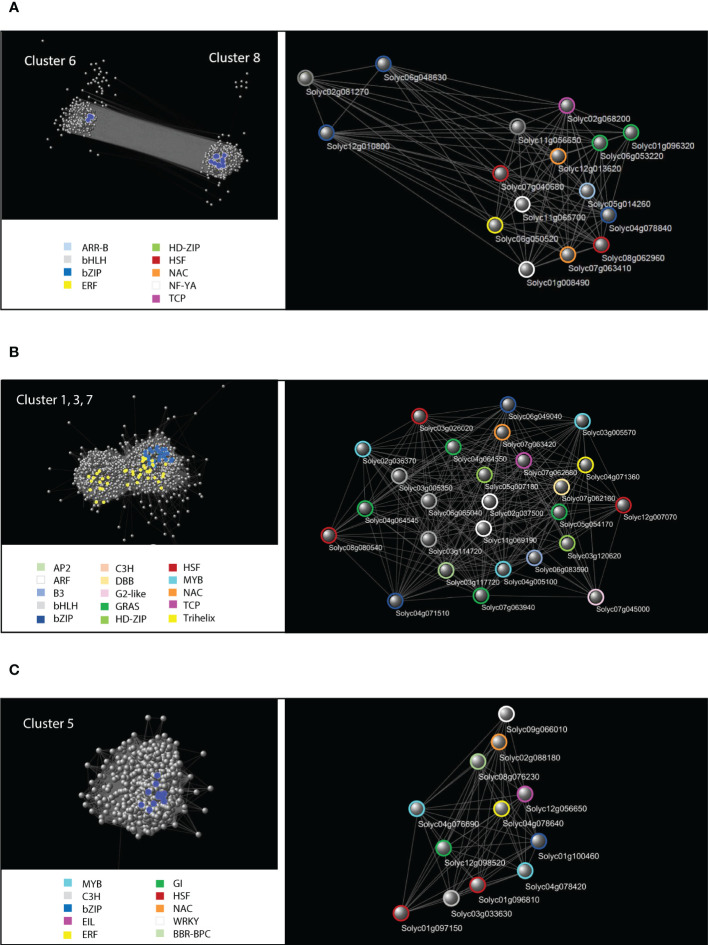
Cluster-specific Gene Co-expression Networks (GCN). **(A)** Cytoscape representation of the GCN constituted by the two anticorrelated clusters 6 and 8 containing genes common to leaf and root osmotic stress response. Upper panel on the left: global GCN with blue spheres representing the TF-encoding DETs falling within the upper 20^th^ percentile of the degree values, which have been detached and magnified in the panel on the right. Colored circles identify specific classes of TFs indicated in the legend. **(B)** Representation of the GCN constituted by the root-specific clusters 1, 3 and 7. Upper panel on the left: global GCN with blue spheres representing the TF-encoding DETs falling within the upper 20^th^ percentile of the degree values, which have been detached and magnified in the panel on the right. Yellow spheres highlight the cell wall genes at the core of the root-specific GCN (**Table 2**). Colored circles identify specific classes of TFs indicated in the legend. **(C)** Representation of the GCN constituted by the leaf-specific cluster 5. Upper panel on the left: global GCN with blue spheres representing the TF-encoding DETs falling within the upper 20^th^ percentile of the degree values, which have been detached and magnified in the panel on the right. Colored circles identify specific classes of TFs indicated in the legend.

To better characterize the transcriptional networks involved in the tomato osmotic stress response, we focused on those groups of genes identified by cluster analysis that showed strong fluctuations in response to the osmotic stress treatment: clusters 6 and 8 represented the common response of leaf and root; clusters 1, 3 and 7 represented the root-specific response; cluster 5 represented the leaf-specific response. Each of these three groups of genes was used to construct and analyze specific GCNs (**Figure 6**). The data related to the network topological analyses are shown in **Table S11**. Hub genes in a GCN are characterized by a high node degree, which is the number of neighbors to which a node directly connects. We considered as hubs all those TFs falling within the upper 20^th^ percentile of the degree values in the topological analysis of the GCN. The identified hub genes that putatively orchestrate osmotic stress response in the three GCNs are listed in **Table 2**.

**Table 2:** List of TF-encoding DETs falling within the 20^th^ percentile of the values of node degree in the three gene co-expression networks.

Accession number	TF family	Cluster	FIGHT CLUB	Gene Name	Function in abiotic stress response	References
TF hubs involved in the common response of leaf and root						
Solyc12g010800.2.1	bZIP	6	–	homologous to *AtbZIP61*	ABA-dependent pathway	Huang et al., 2008 (about Arabidopsis)
Solyc11g056650.2.1	bHLH	8	leaf and root	*SlbHLH96*	ABA-dependent pathway	Liang et al., 2022
Solyc02g068200.1.1	TCP	8	leaf	*SlTP18*	unknown	Parapunova et al., 2014
Solyc11g065700.2.1	NF-YA	8	leaf and root	*/*	unknown	Li et al., 2016
Solyc04g078840.3.1	bZIP	8	leaf and root	*SlAREB1*	ABA-dependent pathway	Orellana et al., 2010; Xu et al., 2022b
Solyc06g053220.3.1	HD-ZIP	8	leaf and root	homologous to *ATHB7*	ABA-dependent pathway	Valdes et al., 2012; A Re et al., 2014 (about Arabidopsis)
Solyc06g050520.3.1	ERF	8	leaf and root	*SlDREB1*	ABA-independent pathway	Thirumalaikumar et al., 2018
Solyc12g013620.2.1	NAC	8	leaf and root	*JA2* (*SlNAC081*)	ABA-JA interaction	Alnemer et al., 2015; Iovieno et al., 2016
Solyc07g063410.3.1	NAC	8	leaf and root	*JA2L* (*SlNAC056/SlRD26*)	ABA-JA interaction	Alnemer et al., 2015; Iovieno et al., 2016
Solyc08g062960.4.1	HSF	8	root	*HsfA2*	thermotolerance	Fragkostefanakis et al., 2019; Hu et al., 2020; Gonzalo et al., 2021
Solyc05g014260.3.1	ARR-B	8	root	homologous to *AtARR11*	ABA-JA interaction	Falconieri et al., 2022 (about Arabidopsis)
Solyc06g048630.3.1	bZIP	6	–	–	unknown	–
Solyc02g081270.4.1	NAC	6	–	–	unknown	–
Solyc07g040680.3.1	HSF	8	root	*HsfA9*	thermotolerance	Fragkostefanakis et al., 2019; Gonzalo et al., 2021
Solyc01g096320.3.1	HD-ZIP	8	leaf and root	homologous to *ATHB12*	ABA-dependent pathway	Valdes et al., 2012; A Re et al., 2014 (about Arabidopsis)
Solyc01g008490.4.1	NF-YA	8	–	–	unknown	Li et al., 2016
TF hubs involved in the root-specific response						
Solyc07g062680.3.1	TCP	7	–	*LANCEOLATE*	unknown	Shleizer-Burko et al., 2011
Solyc07g062160.3.1	DBB	3	root	–	unknown	–
Solyc11g069190.2.1	ARF	7	–	*SlARF4*	drought	Chen et al., 2021
Solyc05g054170.4.1	GRAS	3	root	–	unknown	–
Solyc03g114720.3.1	bHLH	7	–	*SlBIM1a*	unknown	Mori et al., 2021
Solyc03g005570.3.1	MYB	7	–	*AtMIB15*	lignin biosynthesis	Ding et al., 2009 (about Arabidopsis)
Solyc07g063420.3.1	NAC	3	root	*NOR-like1*	unknown	Gao et al., 2018
Solyc05g007180.3.1	HD-ZIP	7	–	homologous to *ATHB13*	drought and salinity response	Cabello and Chan, 2012 (about Arabidopsis)
Solyc04g005100.3.1	MYB_related	7	–	*MYB1R1*	unknown	Aviña-Padilla et al., 2022
Solyc02g037500.1.1	ARF	3	root	–	unknown	–
Solyc04g071510.3.1	bZIP	3	–	–	unknown	–
Solyc03g117720.3.1	AP2	3	root	–	unknown	–
Solyc12g007070.2.1	HSF	3	root	*SlHsfC1*	unknown	–
Solyc06g065040.4.1	bHLH	3	root	*SlbHLH086*	unknown	Sun et al., 2015
Solyc07g063940.2.1	GRAS	3	–	–	unknown	–
Solyc04g071360.4.1	Trihelix	7	–	–	unknown	–
Solyc06g049040.4.1	bZIP	7	–	–	unknown	–
Solyc08g080540.3.1	HSF	3	root	*HSF B-2b*	drought response *via* HY5	Qiu et al., 2019
Solyc03g005350.3.1	bHLH	3	–	*SlbHLH018*	unknown	Sun et al., 2015
Solyc04g064550.1.1	GRAS	3	root	–	unknown	–
Solyc02g036370.3.1	MYB_related	3	root	*SlRVE1*	carotenoid metabolism	Tang et al., 2022
Solyc03g120620.3.1	HD-ZIP	7	–	*SlGL2 (GLABRA2)*	drought response *via* HY5	Qiu et al., 2019
Solyc06g083590.4.1	B3	3	–	*ABI3-2*	unknown	Gao et al., 2013
Solyc03g026020.3.1	HSF	3	–	*HSFB2A*	unknown	–
Solyc07g045000.4.1	G2-like	3	–	–	unknown	–
Solyc04g064545.1.1	GRAS	3	leaf and root	–	unknown	–
Solyc05g006650.3.1	bHLH	7	–	*SlbHLH036*	unknown	Sun et al., 2015
TF hubs involved in the leaf-specific response						
Solyc04g076690.4.1	MYB_related	5	leaf and root	–	unknown	–
Solyc08g076230.1.1	BBR-BPC	5	–	–	unknown	–
Solyc12g056650.2.1	TF	5	leaf	*GIGANTEA2*	unknown	Weraduwage et al., 2015 (about Arabidopsis)
Solyc02g088180.3.1	NAC	5	leaf	*SlORE1S02*	senescence	Lira et al., 2017
Solyc12g098520.2.1	HSF	5	leaf	*HsfA5*	unknown	Fragkostefanakis et al., 2014
Solyc01g097150.4.1	HSF	5	root	–	unknown	–
Solyc04g078640.3.1	ERF	5	leaf	–	unknown	–
Solyc09g066010.3.1	WRKY	5	–	*SlWRKY24*	unknown	Karkute et al., 2018
Solyc01g100460.3.1	bZIP	5	leaf and root	*ABZ1*	ethylene-ABA	Gupta et al., 2022
Solyc03g033630.3.1	C3H	5	leaf	–	unknown	–
Solyc01g096810.3.1	EIL	5	–	*EIL3*	ethylene	Tieman et al., 2001; Gambhir et al., 2022
Solyc04g078420.1.1	MYB	5	leaf and root	*SlMYB70*	ethylene-ABA	Gupta et al., 2022

The “Fight Club” column indicates those genes that have been identified by the SWIM analysis as key genes.

Genes belonging to clusters 6 and 8 formed a GCN which included 1,148 out of the initial 1,159 selected genes (**Figure 6A**). Many genes in the GCN common to leaf and root are involved in stress response, cell cycle reprogramming, secondary metabolism, protein hetero-oligomerization, negative regulation of chromatin condensation, DNA replication, as reported in Revigo analysis of the related clusters (**Table S10**). Five genes encoding core regulatory components of the ABA-mediated signaling pathway were present: *SlPYL2* (Solyc12g055990.2), *SlPP2C1* (Solyc03g096670.3) and *SlPP2C2* (Solyc05g052980.4), *SlPP2C5*/*SlPP2C30* (Solyc03g121880.4), *SlSnRK2.3* (Solyc01g108280.3), *SlSnRK2.8* (Solyc04g012160.4).

Among the 79 TF-encoding DETs present in this GCN, 16 fell within the upper 20^th^ percentile of the values of node degree (**Table 2**) and were considered regulatory hubs of the transcriptional networks that similarly occur in both leaves and roots. Several of these putative hubs are known for their role in ABA-dependent, ABA-independent and ABA-JA crosstalk pathways acting in drought response, whereas eight TFs are not yet uncharacterized for an involvement in stress response (**Table 2**).

The root-related GCN included 2,118 out of the initial 2,147 selected genes belonging to clusters 1, 3 and 7 (**Figure 6B**). Many DETs in the root-related GCN code for proteins and enzymes involved in cell wall metabolism, including arabinogalactan proteins (AGPs), xyloglucan-related enzymes, expansins, glucan endo-1,3-beta-glucosidase, and other proteins with different functions in cell wall remodeling (**Table 3**). The root-related GCN contained 136 TF-encoding DETs, of which 27 were in the upper 20^th^ percentile of the degree values (**Table 2**). Among these TFs, only few have been characterized for their role in abiotic stress, flavonoid metabolism, or lignin biosynthesis, whereas most of them are not yet characterized.

**Table 3:** DETs belonging to the root gene co-expression network that are involved in cell wall formation and remodeling.

Gene function	Accession number
Arabinogalactan proteins (AGPs)	Solyc07g053640.1.1, Solyc10g005960.1.1, Solyc01g091530.4.1, Solyc08g078020.1.1, Solyc01g107340.4.1, Solyc07g065540.1.1
Xyloglucan-related enzymes	Solyc11g065600.2.1, Solyc07g009380.4.1, Solyc08g079040.1.1, Solyc02g092840.1.1, Solyc07g052980.3.1, Solyc09g092520.3.1, Solyc02g080160.4.1, Solyc05g046290.3.1, Solyc07g044960.1.1, Solyc05g005680.3.1, Solyc07g055990.3.1
Expansins	Solyc04g081870.4.1, Solyc06g049050.3.1, Solyc12g089380.2.1, Solyc10g086520.2.1, Solyc06g076220.3.1, Solyc09g010860.4.1, Solyc03g093390.4.1, Solyc06g051800.3.1, Solyc02g088100.3.1
Glucan endo-1,3-beta-glucosidase	Solyc02g080660.3.1, Solyc01g074030.3.1, Solyc08g005000.4.1, Solyc05g025500.3.1, Solyc12g008580.2.1, Solyc04g080260.4.1, Solyc12g040860.2.1, Solyc08g083310.3.1
Other functions in cell wall remodeling	Solyc03g119080.4.1, Solyc08g082250.3.1, Solyc03g111690.4.1, Solyc10g083300.2.1, Solyc08g078020.1.1, Solyc11g005820.1.1, Solyc04g055090.1.1, Solyc06g069430.3.1, Solyc01g107340.4.1, Solyc07g065540.1.1, Solyc09g010090.5.1

The leaf-related GCN included 589 out of the initial 595 selected genes belonging to cluster 5 (**Figure 6C**). This GCN contained 61 TF-encoding DETs, of which 12 fell in the upper 20^th^ percentile of the node degree values (**Table 2**). Even in this case, only few TFs were already described, and were mainly involved in senescence and ethylene-ABA crosstalk, whereas 6 TFs were uncharacterized and may represent interesting new players in water stress response.

The SWIM computational analysis is able to identify genes (named “switch genes”) with a critical role in a transcriptional network. In our study, this analysis defined most of the hub TFs of the three GCNs as “FIGHT CLUB” genes, which are characterized by a marked negative correlation with their first nearest neighbors (**Table 2**). As shown in **Figure 6**, these TF-encoding DETs are placed at the core of the GCNs and are connected with most of the other nodes, suggesting a pivotal role in the response to osmotic stress in both leaf and root and their possible role as transcriptional repressors.

### 3.6 Specific response of M82 and Tondo genotypes to osmotic stress

**Figure 7:**
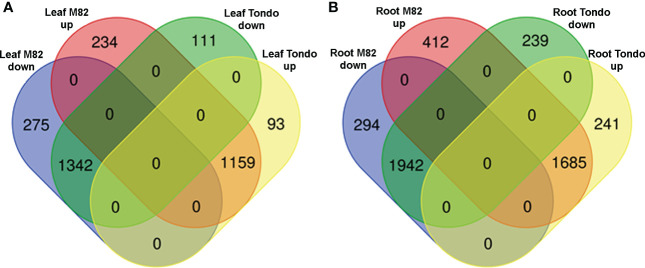
Venn diagram of the DETs specifically regulated in M82 and Tondo leaf **(A)** and root **(B)**.

As reported in **Figure 2**, a minor component of the transcriptomic response to the osmotic stress resulted genotype-specific. The number of specific DETs was higher in M82 than in Tondo (1,215 *vs* 684; **Tablse 1**, **S12**). In M82 leaf, 234 and 275 transcripts were up- and down-regulated, respectively, whereas in Tondo leaf 93 and 111, respectively. In M82 roots, we found 412 up-regulated transcripts and 294 down-regulated transcripts, whereas in Tondo roots 241 and 239. (**Figure 7**). Regarding TF-encoding DETs, 51 resulted genotype-specific in leaf (37 in M82 and 14 in Tondo), whereas 103 resulted genotype-specific in root (61 in M82 and 42 in Tondo; **Table S11**).

We then searched those genes (both DETs and non-DETs) showing a very different expression level between the two genotypes (*i.e.*, “M82 control/Tondo control” ≥ |2|), under non-stressed conditions, and 15 genes met this criterion (**Figure S4**). In leaf, four genes were detected (**Figure S4A**), including *ASN1* (Solyc06g007180.3), that encodes an asparagine synthetase, and *SlE8* (Solyc09g089580.4), a negative regulator of ethylene biosynthesis. In root, seven genes were detected with different basal expression level between M82 and Tondo (**Figure S4C**), including an adenylate kinase (ADK) encoding gene (*ADK11*, Solyc12g010380.3), and a photosystem-related gene (Solyc06g084050.4). Finally, we observed four genes with different basal expression level in both leaf and root between the two genotypes (**Figure S4B**), three of which code for TFs: the HD-ZIP *SlANL2b* (Solyc06g035940.3), and two MADS-box TFs belonging to the AGAMOUS subfamily (Solyc07g052700.3 and Solyc07g052720.4).

## 4 Discussion

The aim of the present study was to provide a detailed picture of the transcriptomic response to osmotic stress in tomato, and to shed light on the stress-related regulatory pathways occurring in leaves and roots. For this purpose, tomato plants of the genotypes M82 and Tondo were subjected to a PEG-mediated osmotic treatment. The M82 genotype was selected since it is reported as a drought-sensitive genotype (Gong et al., 2010; Liu et al., 2017; Li et al., 2018a) and has been previously characterized for its response to water stress (Iovieno et al., 2016; Liu et al., 2017). The Italian cultivar Tondo, with typical round-fruits used in the industrial tomato sector, was selected amongst a small panel of Italian genotypes for transcript profiling upon osmotic stress in addition to the well characterized M82 genotype.

Treatment with high molecular weight PEG has been widely used in hydroponic systems to mimic plant water deficit *via* osmotic effects, with minimal toxic effects on plants (Verslues et al., 2006). PEG exposure allowed to study the effect of osmotic condition on various cellular and physiological parameters, such as leaf gas exchange rates, plant water potentials, antioxidative response, as well as on molecular mechanisms and gene expression (Li et al., 2012; D’Amico-Damião et al., 2021; Li et al., 2022; Zhao et al., 2023). Drought is a complex stressful condition for plants and PEG-induced osmotic stress mimics one specific component, since moderate or severe drought determines the accumulation of salts and ions in the soil leading to osmotic stress. The comparison of our transcriptomic data with those collected during medium/severe drought by other authors (Ferrández-Ayela et al., 2016; Iovieno et al., 2016; Liu et al., 2017; Zhou et al., 2019) indicated that the transcriptional changes induced by osmotic stress during water deprivation play a prominent role in the alteration of transcriptional response. Under osmotic stress, initial stomatal closure and root-to-shoot signaling can occur within 1 h, triggering osmotic adjustment associated with increased solute accumulation (Dubois and Inzé, 2020). Therefore, short-term osmotic stress induced by PEG can be useful to discover gene expression changes related to water stress response.

Our transcriptomic analysis detected a total of more than six thousand DETs in response to the PEG treatment, with the more sensitive M82 genotype showing a slightly higher number of DETs than the less sensitive Tondo (**Table 1**). The transcriptional response to osmotic stress was largely shared by the two genotypes in both leaves and roots (**Table 1** and **Figure 2**). Regarding this transcriptional response, GCNs related to the leaf and root common response, the root-specific and the leaf-specific responses were analyzed to identify the molecular pathways involved in the stress response and the TF genes acting as hubs in these regulatory networks.

### 4.1 Characterization of the gene co-expression network common to leaf and root and identification of putative hub transcription factors

ABA plays a key role in plant adaptation to adverse environmental conditions (Zhang et al., 2022). The induction of ABA synthesis is one of the most rapid phytohormonal responses of plants to abiotic stresses, thereby triggering ABA-inducible gene expression, causing stomatal closure, and hence reducing water loss *via* transpiration, which will eventually restrict cellular growth (Wilkinson and Davies, 2010). Our data confirmed the upstream role of ABA signaling in the response to osmotic stress in the whole plant. Indeed, the gene 9-cis-epoxycarotenoid dioxygenase (*NCED1*, Solyc07g056570.1), a rate limiting enzyme of ABA biosynthesis able to improve tolerance to dehydration stress when overexpressed in plants (Iuchi et al., 2001), was induced by the PEG treatment in both leaves and roots and placed in cluster 8. The ABA signaling transduction pathway includes four core regulatory components: ABA receptor/pyrabactin resistance protein1/PYR-like protein (RCARs/PYR1/PYLs), protein phosphatases type 2C (PP2C), the sucrose nonfermenting1-related protein kinase 2 (SnRK2) and ABA responsive element binding factors (ABF; Ma et al., 2009). The ABA-related genes *SlPYL2*, *SlPP2C1* and *SlPP2C2*, *SlPP2C5*/*SlPP2C30*, *SlSnRK2.3*, *SlSnRK2.8* were present in the leaf-root GCN (Chen et al., 2016; Krukowski et al., 2023). The gene *SlPP2C5*/*SlPP2C30* was found to be regulated by ABA treatment by Zhang and colleagues (2019). In addition, five TF genes identified as hubs are involved in the ABA-dependent signaling pathways (**Table 2**). The ABF *SlAREB1* (Solyc04g078840.3) is well characterized for its involvement in the ABA-mediated stress response pathway and for conferring tolerance when overexpressed (Orellana et al., 2010; Zhang et al., 2019; Xu et al., 2022b). A further bZIP gene (Solyc12g010800.2), that showed the highest degree in the GCN, is the putative ortholog of *AtbZIP61*, known to be involved in abiotic stress response (Oono et al., 2006; Huang et al., 2008). It is noteworthy that two hub TFs (Solyc06g053220.3 and Solyc01g096320) are the putative orthologs of Arabidopsis *ATHB7* and *ATHB12*, that are involved in water and salinity stress and modulate abscisic acid signaling by regulating the PP2Cs and the PYR/PYL family of ABA receptors (Valdés et al., 2012;. Ré et al., 2014). *ATHB7* and *ATHB12* tomato orthologs may play similar upstream roles in the tradeoff between plant development and water stress response. Finally, *SlbHLH96* (Solyc11g056650.2), with a high degree in the GCN, was previously characterized for its ability to confer drought tolerance in an ABA-dependent manner, through the repression of the ABA catabolic gene *SlCYP707A2* (Liang et al., 2022).

Recent studies underlined the strong interconnection between ABA and JA signaling pathways during the abiotic stress response (Riemann et al., 2015; de Ollas and Dodd, 2016; Buti et al., 2019). The identified NAC hub genes *JA2* (Solyc12g013620.2, also named *SlNAC081*) and *JA2L* (Solyc07g063410.3, also named *SlNAC056/SlRD26*) are induced by ABA, promote stomatal closure, and are involved in the reprogramming of the physiological responses during drought and salt stress response acting in the JA signaling pathway (Du et al., 2014; Alnemer, 2015; Iovieno et al., 2016; Liu et al., 2019; Martínez-Andújar et al., 2020). The ARR-B hub gene (Solyc05g014260.3) may also be involved in JA signaling, as the Arabidopsis homolog *RESPONSE REGULATOR 11* (*ARR11*) affects ABA-JA crosstalk (Falconieri et al., 2022).

In response to abiotic stress, the ABA-independent pathway has been also well characterized in many plant species (Shinozaki and Yamaguchi-Shinozaki, 2007; Yoshida et al., 2014). In the leaf-root GCN, the ERF TF gene *SlDREB1* (Solyc06g050520.3) plays a role in drought and osmotic stress tolerance in the ABA-independent pathway (Thirumalaikumar et al., 2018; Kumar et al., 2019). Finally, two heat shock factors, *HsfA2* (Solyc08g062960.4) and *HsfA9* (Solyc07g040680.3), that showed a high degree in the GCN (**Table 2**), are known to have an important role in tomato thermotolerance (Fragkostefanakis et al., 2019; Hu et al., 2020; Gonzalo et al., 2021). Hence, a possible role of these genes in osmotic stress tolerance is plausible.

Overall, these data confirmed that the ABA-dependent and ABA-independent pathways, as well as the cross-talk with the JA signaling pathway, are crucial for the plant water stress response in the whole plant. Based on the so-called “guilt by association” principle, the other putative hubs identified as strictly connected with these known regulatory networks may represent novel players in the ABA-mediated and ABA-JA crosstalk during water stress.

### 4.2 Characterization of the root-specific gene co-expression network and identification of putative hub transcription factors

Root is the first organ that perceives water or osmotic stress, and the stress signal moves from roots to leaves through cell to cell signaling networks (Janiak et al., 2016). For this reason, the stress sensing and consequent response in root play a major role in tolerance. Water stress response involves a restructuring of the cell wall that allows growth processes to occur at a lower water level; thus, cell wall adjustment under osmotic stress is an important phenomenon in plant adaptation, as previously observed (Jiang and Deyholos, 2006; Moore et al., 2008; Baldoni et al., 2016). Consistently, many DETs in the root-related GCN are involved in cell wall metabolism with different functions in cell wall remodeling (**Table 3**). Among them, two fasciclin-like AGPs (*FLAs*) genes (Solyc10g005960.1 and Solyc01g091530.4), have previously been described for their role in the cell wall remodeling during water stress (Veronico et al., 2022).

Among the hub genes in the root GCN, *TM4/TDR4/FULL/FUL1* (Solyc06g069430.3), *LANCEOLATE* (Solyc07g062680.3), and *NOR-like1* (Solyc07g063420.3) encode important TFs involved in different processes in tomato. The MADS box *TM4/TDR4/FULL/FUL1*, is known to be repressed by LANCEOLATE that play a role in the promotion of leaf differentiation (Shleizer-Burko et al., 2011; Burko et al., 2013). The *TM4/TDR4/FULL/FUL1* expression is also influenced by NOR-like1. NOR-like1 is involved in fruit ripening directly influencing the expression of many genes, some of which involved in cell wall metabolism and found in our root-related GCN: *CEL2* (Solyc09g010210.3), *CEL8* (Solyc08g082250.3), *EXP1* (Solyc06g051800.3), and *PL* (Solyc03g111690.4; Gao et al., 2018). Our data suggest that these genes could be involved in a gene regulatory module of cell wall remodeling in root under osmotic stress. In addition, the hub TF SlBIM1a, regulates cell expansion through the repression of an expansin (Solyc02g088100.3), which is present in our root-related GCN (Mori et al., 2021).

Secondary metabolism plays a key role in the response to water stress, through ROS scavenging and cell wall remodeling and MYB genes have a prominent role in the related regulatory networks (Baldoni et al., 2015; Yadav et al., 2021; Xu et al., 2022a). Two MYB hub TFs were found in the root GCN: MYB-related SlRVE1 (Solyc02g036370.3), involved in carotenoid metabolism (Tang et al., 2022), and the putative ortholog of ATMYB15 (Solyc03g005570.3), activating lignin biosynthetic genes and involved in stress response (Ding et al., 2009; Baldoni et al., 2013). In addition, the hub TF SlARF4 (Solyc11g069190.2) is involved in ABA-mediated drought response through the interaction with SlMYB72 (Wu et al., 2020; Chen et al., 2021).

Two more hub TFs, HY5 (Solyc08g061130.3) and an HSF (Solyc12g007070.2), play a key role in controlling anthocyanin accumulation in response to light (Liu et al., 2018; Qiu et al., 2019). In a recent transcriptomic analysis related to drought response, HY5 has been identified as a pivotal TF, directly interacting with the HSF TF gene *B-2b* (Solyc08g080540.3) and *SlGL2* (Solyc03g120620.3; Qiu et al., 2019). These two TFs also played a prominent role in the root-related GCN (**Table 2**). *SlGL2* is the putative ortholog of the Arabidopsis gene *AtGL2*, a well-characterized regulator of epidermal cell differentiation (Tominaga-Wada et al., 2009; Lashbrooke et al., 2015). Hence, these four TFs may represent a gene regulatory module in the response to osmotic stress in root.

These identified hub TFs might be involved in the switch from developmental programs to stress response. In literature, few information is available on the function of TFs in tomato root since most of the studies in tomato are related to leaf or fruit development. Here, several uncharacterized TFs were found in the root-specific regulatory network that may play a role in osmotic stress, including four bHLH, four GRAS and three HSF TFs. Our results can help in shedding new light in the gene regulatory networks controlling cell wall remodeling and secondary metabolism during osmotic stress response in tomato roots.

Strikingly, in root we also found up-regulation of photosynthesis-related genes. This is consistent with previous findings on rice (Minh-Thu et al., 2013; Baldoni et al., 2016). In particular, Minh-Thu and colleagues (2013) found that specific photosynthetic genes showed an opposite expression pattern in leaf and root and observed a clear stress-related induction in root also under dark condition, confirming a tissue-specific regulation of these photosynthetic genes during dehydration stress.

### 4.3 Characterization of the leaf-specific gene co-expression network and identification of putative hub transcription factors

A crucial point of water stress response in leaf is the balance between leaf senescence and the maintenance of photosynthesis Fine-tuning of this balance through the modulation of gene expression by specific TFs can be the key to improve tolerance (Jan et al., 2019; Baldoni et al., 2021). It is known that leaf senescence is activated by abiotic stresses, including drought, through an ABA-mediated mechanism (Takasaki et al., 2015; Mao et al., 2017). During leaf senescence, several morphological, physiological, and molecular changes occur. In particular, photosynthesis is down-regulated whereas nitrogen remobilization is up-regulated. Senescence-related genes in cluster 5 and in the leaf-related GCN were up-regulated in response to osmotic stress. Differently, many photosynthesis-related genes of cluster 2 were down-regulated in leaves in response to stress.

The NAC TF *SlORE1S02* (Solyc02g088180.3), one of the TFs with the highest degree in the leaf-specific GCN (**Table 2**), is involved in leaf senescence (Lira et al., 2017). Its Arabidopsis ortholog, *AtORE1*, encodes a master regulator of senescence initiation (Rauf et al., 2013; Garapati et al., 2015). Other TFs belonging to the leaf GCN are involved in leaf senescence: the NAC *SlNAP1* (Solyc05g007770.3; Ma et al., 2018), a putative senescence associated gene (Solyc01g104080.4) and the hexokinase *SlHXK3* (Solyc12g008510.2). Hexokinase are involved in the changes in sugar metabolism that act during senescence. In particular, *SlHXK3* seems to be involved in the maintenance of hexokinase activity and integrity of mitochondrial functions in young and mature leaves, as well as in production of ROS by mitochondrial electron transport (Poór et al., 2018, 2019).

ABA and ethylene are commonly recognized to act antagonistically in the control of plant growth and development, although positive interactions between these two hormones have also been shown (Müller, 2021). Interestingly, the two hub TFs *ABZ1* (Solyc01g100460.3) and *SlMYB70* (Solyc04g078420.1) are part of the network of *SlDREB3*, a negative regulator of ABA responses, and seem to be connected within an ethylene-mediated signaling pathway and (Gupta et al., 2022). In particular, SlMYB70 is a negative regulator of ethylene biosynthesis and fruit ripening (Cao et al., 2020). In addition, the TF ETHYLENE INSENSITIVE3-like *EIL3* (Solyc01g096810.3) is a positive regulator of ethylene response (Tieman et al., 2001; Gambhir et al., 2022). The characterization of the TFs with unknown role identified as hubs in the leaf-specific GCN, and highly co-expressed with the TFs in the ABA-ethylene crosstalk, may contribute to better dissect the role of this regulatory module in the tomato leaf response to osmotic stress.

### 4.4 Genotype-specific genes involved in the osmotic stress response

Although most DETs were commonly regulated by the stress treatment in both genotypes, a genotype-specific response was present, more consistent in root than in leaf and in M82 than in Tondo. These genes may be responsible of the differences in the response to osmotic stress of M82 and Tondo, being M82 slightly but significantly more sensitive to the osmotic treatment than Tondo.

In root, we detected 103 TFs differently regulated between the two genotypes. Among them, we found two TFs involved in leaf senescence, as *SlWRKY53* (Solyc08g008280.3) and *SlERF.F5* (Solyc10g009110.1; Chen et al., 2022; Wang et al., 2022), and two TFs involved in chlorophyll biosynthesis, as *SlBL4* (Solyc08g065420.3) and *SlBEL11* (Solyc11g068950.3; Meng et al., 2018; Yan et al., 2020). A function in abiotic stress response was previously hypothesized for these two genes (He et al., 2022). It is not clear why these genes, that are involved in processes related to leaf metabolism, showed a stress-related regulation in roots. Nevertheless, these data and the observed regulation of photosynthetic genes in root during water or osmotic stress highlighted the importance of the interconnection between the molecular stress response of leaf and root (Minh-Thu et al., 2013; Baldoni et al., 2016).

Fine tuning of lignin biosynthesis for cell wall formation and of flavonoids production for defense against ROS damage might influence stress response (Xu et al., 2022a). Three TF-encoding genes involved in these processes resulted differently regulated in the two tomato genotypes. The *MYB* gene *SlTHM27* (Solyc10g055410.2) is the tomato orthologue of *AtMYB4*, involved in the regulation of the phenylpropanoid pathway (Adato et al., 2009). The *MYB* gene *SlMIXTA-like* is involved in phenylpropanoid and flavonoid metabolic pathways and in cell wall formation (Lashbrooke et al., 2015; Ye et al., 2015). The HD-ZIP *SlANL2b* (Solyc06g035940.3) is involved in cell wall formation and cuticle assembly (Shi et al., 2021). Other three TFs, *SlNAC1* (Solyc04g009440.3), Solyc03g124110 and *CBF1* (Solyc03g026280.3), are involved in abiotic stress response (Wang et al., 2016). Interestingly, *CBF1* resulted differently regulated in a comparative transcriptomic study between two genotypes with contrasting cold stress response (Albenga, cold-tolerant, and San Marzano, cold-sensitive), suggesting its potential in influencing abiotic stress response (Caffagni et al., 2014).

In leaf, among the 51 TF DETs with genotypic-specific expression, three genes may play an important role in the osmotic stress response of the two genotypes: a *MYB* gene (Solyc01g102340.3) involved in cell wall differentiation (Huang et al., 2017), and *SlWRKY81* (Solyc09g015770.3) and *SlZF3/ZAT12* (Solyc06g075780.3) known to be involved in tolerance to high salinity (Hichri et al., 2017; Li et al., 2018b; Martínez-Andújar et al., 2020).

In response to environmental constraints, differences in the basal expression level of specific genes can make plants more or less prepared to counteract the extreme conditions. For this reason, we searched for those genes showing a different expression level in control samples of M82 and Tondo.

In leaf, *ASN1* may have an important role in osmotic stress response, since asparagine synthetase is involved in sensing and signaling of nutritional status during the early response to abiotic stresses (Gaufichon et al., 2010). *SlE8*, which is a negative regulator of ethylene biosynthesis, may be involved in ethylene signaling pathway (Van de Poel et al., 2014). ADK is a crucial enzyme in maintaining energy metabolism and in plants is involved in abiotic stress responses (Yang et al., 2021). Interestingly, an *ADK* gene was induced by drought stress in a drought-tolerant tomato genotype (Gong et al., 2010), suggesting a role for *ADK11* in differentiating the response of Tondo and M82.

Finally, we considered the three TF genes with different basal expression level between the two genotypes in both leaf and root (**Figure S4B**). *SlANL2b*, that was above mentioned for its exclusive down-regulation in Tondo, showed a higher basal expression in M82, confirming its different behavior between the two genotypes. Two MADS-box TFs belonging to the AGAMOUS subfamily, showing higher expression in Tondo than in M82 in control conditions, play key roles in the development and determination of reproductive floral organs (Dreni and Kater, 2014). Interestingly, a recent study identified a tomato *AGAMOUS* gene that confers tolerance to salt stress (Guo et al., 2016). Further analyses are needed to assess whether these genes may be responsible of the different sensitivity to osmotic stress in these genotypes.

## 5 Conclusions

**Figure 8:**
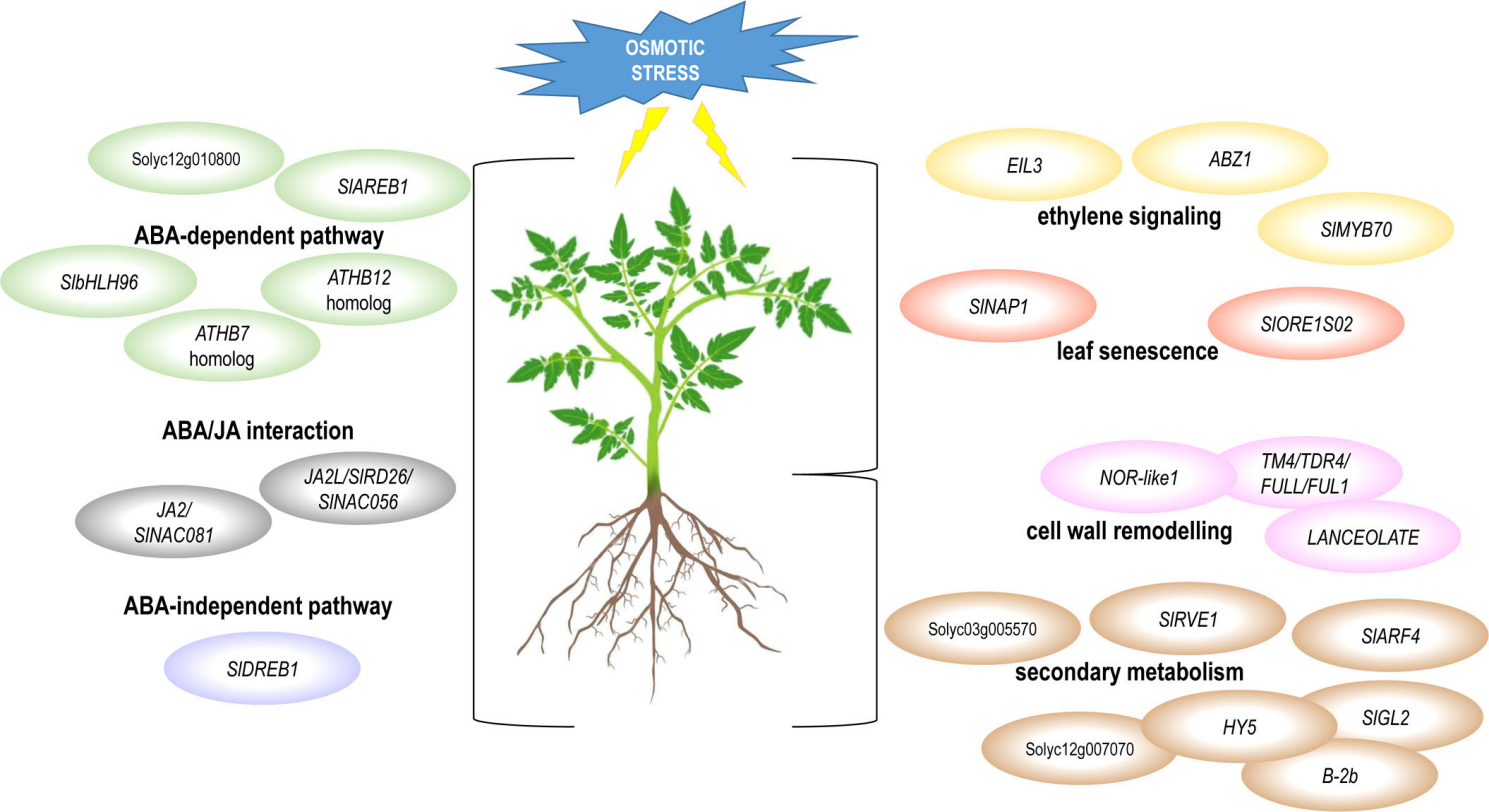
Graphical representation of the signaling or metabolic pathways involved in the response to osmotic stress in tomato. The pathways that resulted common or specific to leaf and root, as well as the identified TF-encoding DETs, that have been already described for their involvement in these pathways, are represented.

This study provides a comprehensive overview of the stress-related transcriptome changes that occur in both roots and leaves of two tomato genotypes in response to osmotic stress. The transcriptomic landscapes of the common and the specific response of leaf and root have been described, and the gene regulatory pathways operating in these plant organs were dissected. The common response of leaf and root was characterized by ABA-dependent, ABA-independent and JA signaling pathways. The root-specific response included genes involved in cell wall remodeling and secondary metabolism, whereas the leaf-specific response was related to leaf senescence and ethylene signaling. The proposed models are presented in **Figure 8**. Among the hub genes of these regulatory networks (**Table 2**), we identified TF-encoding DETs as hubs already known for their involvement in abiotic stress response, whereas several TF-encoding genes have not yet characterized or linked to stress response in tomato. These novel TF hub genes could be important regulators of osmotic stress response in tomato and should be further analyzed and validated by functional studies to dissect their role in stress response.

## Data availability statement

The original contributions presented in the study are publicly available. This data can be found here: GEO accession GSE224629.

## Author contributions

AG, EB, RP, and GF conceived and designed the research. AG, EB, FL, and MM performed stress experiments and physiological analysis. EB performed gene expression analysis. RP and GF performed bioinformatic analyses. EB, RP, and GF analyzed the data. EB wrote the manuscript. RP and GF contributed to wrote the manuscript. All authors read and approved the manuscript. All authors contributed to the article and approved the submitted version.

## Supplementary material

Supplementary Figure 1 Principal component analysis of the differential expression data related to the 24 leaf and root samples of M82 and Tondo under control and osmotic stress conditions. LT_C: Tondo leaves, control. LT_P: Tondo leaves, PEG-treated. LM_C: M82 leaves, control. LM_P: M82 leaves, PEG-treated. RT_C: Tondo roots, control. RT_P: Tondo roots, PEG-treated. RM_C: M82 roots, control; RM_P: M82 roots, PEG-treated.

Supplementary Figure 2 Validation of the expression of selected genes from microarray using qRT-PCR. Fold changes in gene expression were transformed to a log2 scale. The microarray log2 values (X-axis) were plotted against the qRT-PCR data log2 values (Y-axis). The function of the regression line and the R^2^ value are shown.

Supplementary Figure 3 Venn diagram showing the distribution of the 439 identified TF-encoding DET genes as up-or down-regulated in leaf (L) and root (R) microarrays in each of the comparisons.

Supplementary Figure 4 Whisker box plot of microarray expression values of the genes differently expressed in the control samples of M82 *vs* Tondo in the three biological replicates (“M82 control/Tondo control” ≥ |2|). The ends of the box represent the upper and lower quartiles, so the box spans the interquartile range. The median is marked by a horizontal black line inside the box. Statistical significance was determined by Student’s t–test between either root or leaf microarray expression values. Statistically significant differences are indicated with an asterisk (* p ≤ 0.05; ** p ≤ 0.01; *** p ≤ 0.001) for those genes where the ratio “M82 control/Tondo control” was ≥ |2|. **(A)** Genotype specific genes differentially expressed in the leaf only. **(B)** Genotype specific genes differentially expressed in both leaf and root. **(C)** Genotype specific genes differentially expressed in the root only.

Supplementary Table 1 Raw data derived from the hybridization of the NimbleGen microarray “111012_Slyc_PT_expr”. The data are referred to the original 28,191 tentative consensus (TC) sequences and to the related 16,654 Solyc transcripts from ITAG 4.0. The correspondence of TC and Solyc IDs is reported.

Supplementary Table 2 List of differentially expressed transcripts (control *vs* PEG) in the four considered comparisons (Leaf M82, Leaf Tondo, Root M82, Root Tondo).

Supplementary Table 3 AgriGOv2 and Revigo analysis reports on leaf and root DETs.

Supplementary Table 4 K-means cluster analysis of DETs. The cluster score is reported.

Supplementary Table 5 SWIM analysis.

Supplementary Table 6 Selected genes and primers used for qRT-PCR analysis.

Supplementary Table 7 Physiological measurements of tomato plants in preliminary experiments for osmotic stress. Comparisons of differences between control and treated plants were performed with Student’s *t*-tests. **(A)** Comparison among 5, 10, 20% PEG. **(B)** Comparison between 3 and 24 h at 20% PEG. **(C)** Comparison among the 5 genotypes at 20% PEG for 24 h; different letters in the same column show significant differences based on a Tukey’s test (*p* ≤ 0.001).

Supplementary Table 8 Log2 Fold Change of selected DETs from microarray and qRT-PCR data used for validation of gene expression data.

Supplementary Table 9 List of the 439 TF-encoding DETs. The Log2 Fold Change data, the belonging to the TF families and the number of the TF up- or down-regulated in leaf and roots are reported.

Supplementary Table 10 AgriGOv2 and Revigo analysis results of the eight clusters.

Supplementary Table 11 Network analysis statistics of the gene leaf and root, root-specific, and leaf-specific co-expression networks created using Pearson’s correlation value |r| ≥ 0.8. The leaf and root GCN included 1148 genes belonging to clusters 6 and 8 with a cluster score ≥ 0.9. The root-specific GCN included 2118 genes belonging to clusters 1, 3 and 7 with a cluster score ≥ 0.9. The leaf-specific GCN included 589 genes belonging to cluster 5 with a cluster score ≥ 0.9.

Supplementary Table 12 List of the differentially regulated transcripts (DETs) that are specifically regulated under osmotic stress in M82 and Tondo.
