# Supplementary figures and images for: Transcriptomic analysis reveals the gene regulatory networks involved in leaf and root response to osmotic stress in tomato
Source: Front Plant Sci. 2023 Jun 2;14:1155797. doi: 10.3389/fpls.2023.1155797 (PMC10272567; doi:10.3389/fpls.2023.1155797)

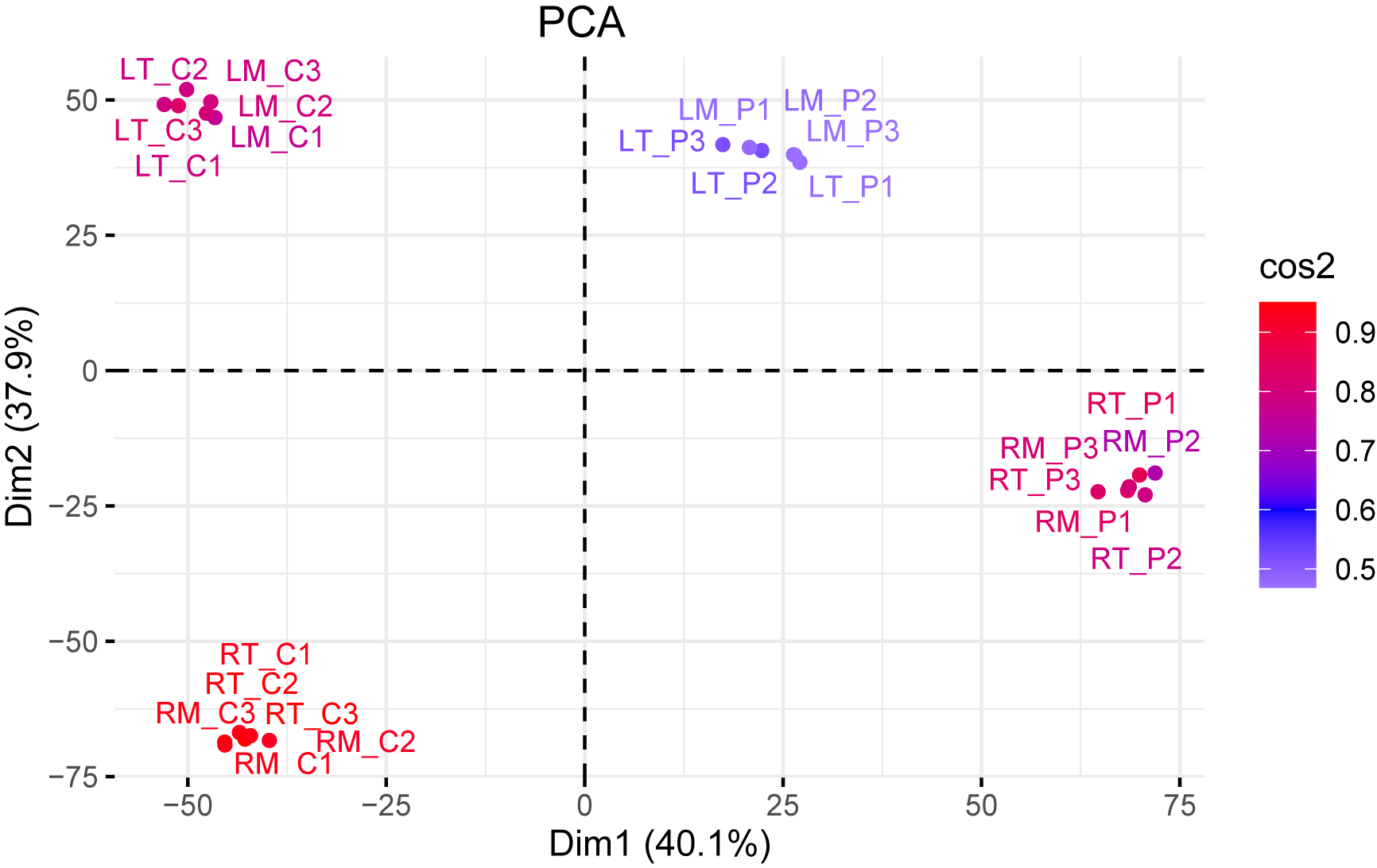

Supplement: Supplementary Figure 1 — Principal component analysis of the differential expression data related to the 24 leaf and root samples of M82 and Tondo under control and osmotic stress conditions. LT_C: Tondo leaves, control. LT_P: Tondo leaves, PEG-treated. LM_C: M82 leaves, control. LM_P: M82 leaves, PEG-treated. RT_C: Tondo roots, control. RT_P: Tondo roots, PEG-treated. RM_C: M82 roots, control; RM_P: M82 roots, PEG-treated. [file Image_1.tif]

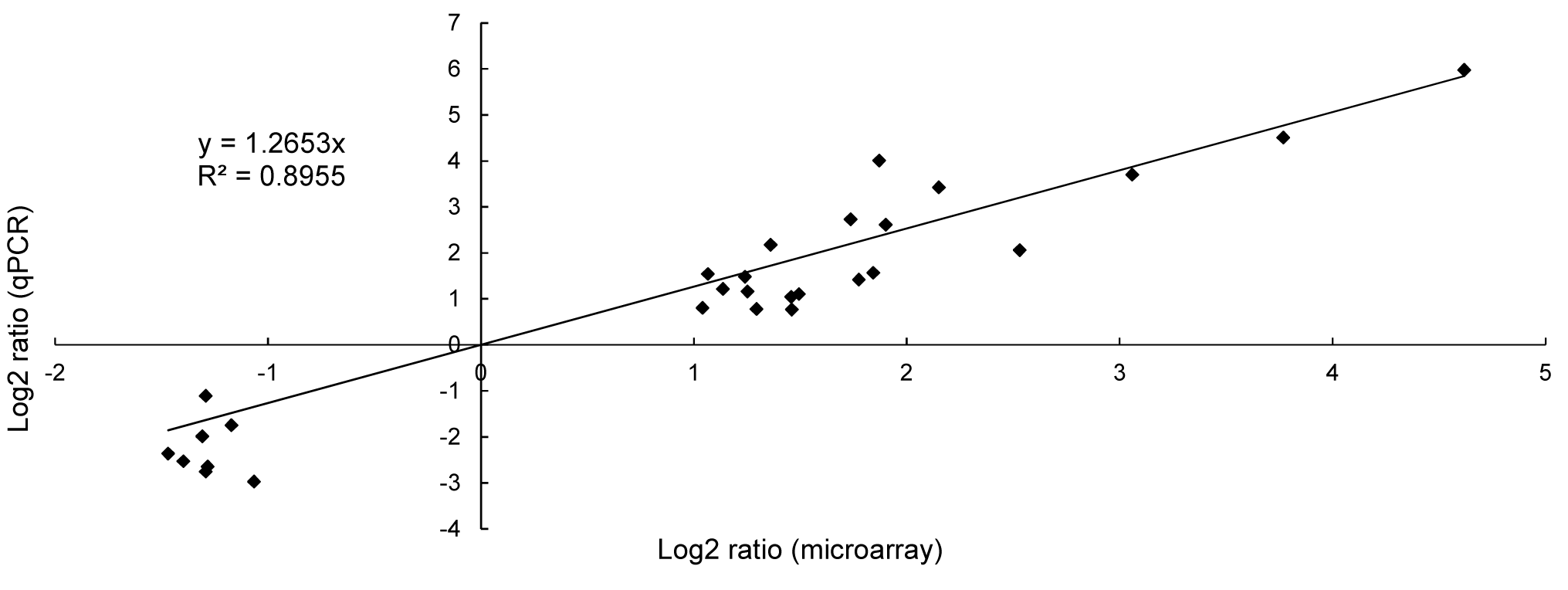

Supplement: Supplementary Figure 2 — Validation of the expression of selected genes from microarray using qRT-PCR. Fold changes in gene expression were transformed to a log2 scale. The microarray log2 values (X-axis) were plotted against the qRT-PCR data log2 values (Y-axis). The function of the regression line and the R2 value are shown. [file Image_2.tif]

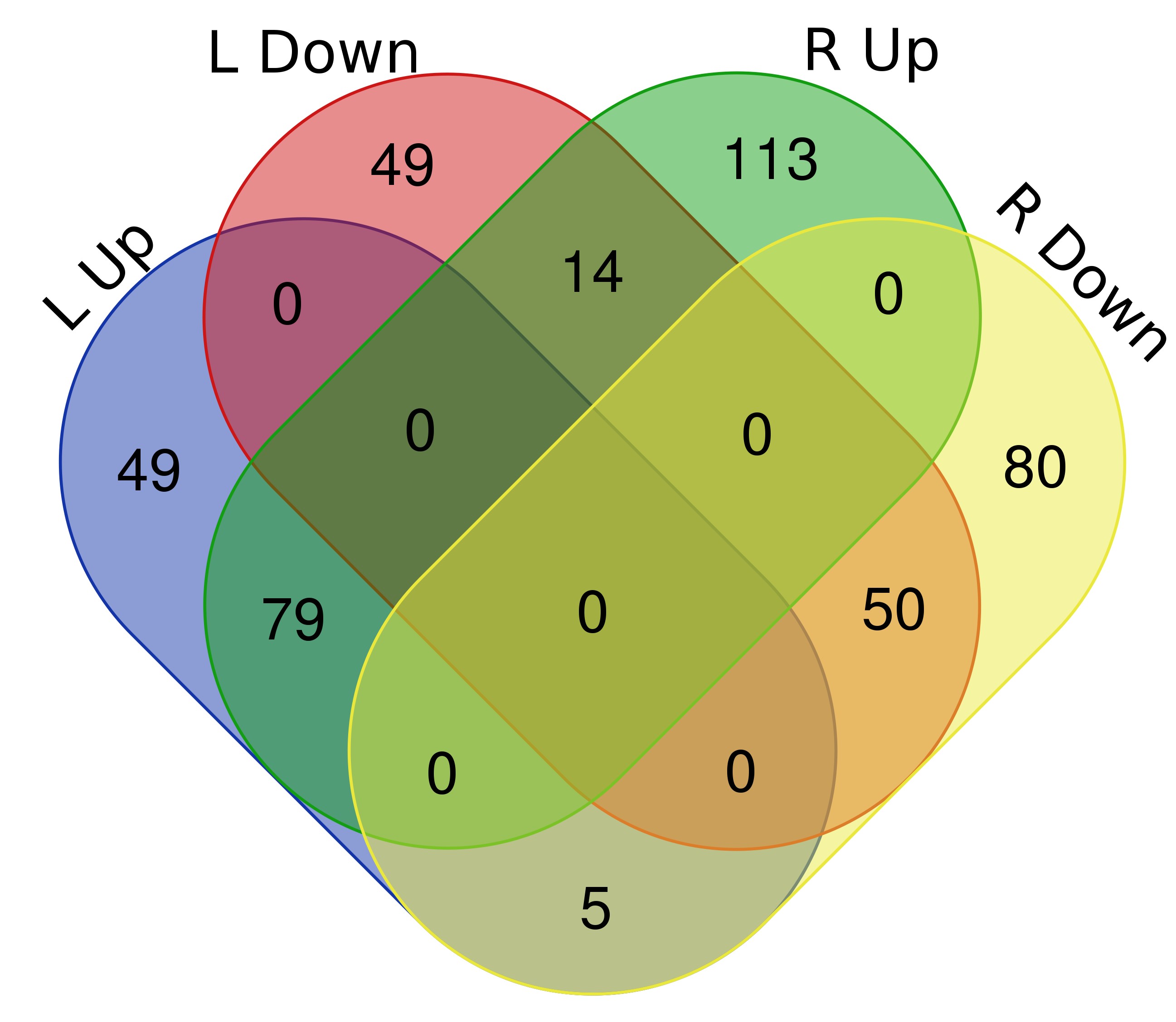

Supplement: Supplementary Figure 3 — Venn diagram showing the distribution of the 439 identified TF-encoding DET genes as up-or down-regulated in leaf (L) and root (R) microarrays in each of the comparisons. [file Image_3.jpeg]

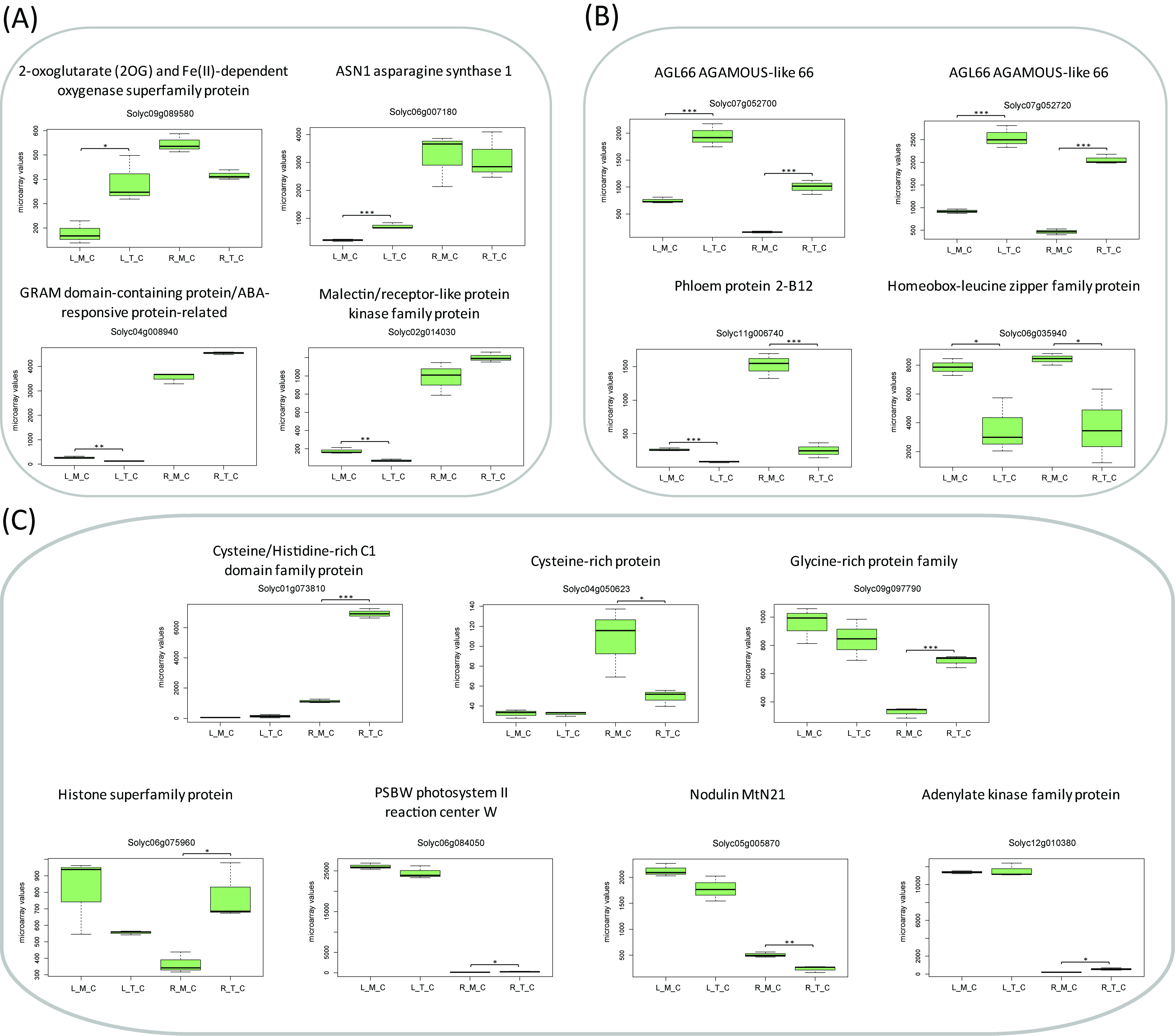

Supplement: Supplementary Figure 4 — Whisker box plot of microarray expression values of the genes differently expressed in the control samples of M82 vs Tondo in the three biological replicates (“M82 control/Tondo control” ≥ |2|). The ends of the box represent the upper and lower quartiles, so the box spans the interquartile range. The median is marked by a horizontal black line inside the box. Statistical significance was determined by Student’s t–test between either root or leaf microarray expression values. Statistically significant differences are indicated with an asterisk (* p ≤ 0.05; ** p ≤ 0.01; *** p ≤ 0.001) for those genes where the ratio “M82 control/Tondo control” was ≥ |2|. (A) Genotype specific genes differentially expressed in the leaf only. (B) Genotype specific genes differentially expressed in both leaf and root. (C) Genotype specific genes differentially expressed in the root only. [file Image_4.tif]
