# Supplementary material for: Transcriptomic analysis reveals the gene regulatory networks involved in leaf and root response to osmotic stress in tomato
Source: Front Plant Sci. 2023 Jun 2;14:1155797. doi: 10.3389/fpls.2023.1155797 (PMC10272567; doi:10.3389/fpls.2023.1155797)
Supplement: Supplementary Table 6 — Selected genes and primers used for qRT-PCR analysis. [file Table_6.docx]

Table S6: Selected genes and primers used for qRT-PCR analysis.

| **TC** | **SOLYC ID** | **Primer sequences** |
| --- | --- | --- |
| TC217348 | Solyc07g056670 | For 5’-GAATCACTATCCGCCATGCT-3’  Rev 5’-GGTGGGACAGAGATCCAGTG-3’ |
| TC219605 | Solyc07g052700 | For 5’-GGAAAATGCCAAACTGGAGA-3’  Rev 5’-CACCCATTGGAAGTTGGACT-3’ |
| TC230591 | Solyc12g010380 | For 5’-ATATCCCGGAGCTGAATCCT-3’  Rev 5’-ACCTTGACACGCTTTCCATC-3’ |
| TC235927 | Solyc10g017990 | For 5’-CATATTGCAGCCAAAGGTCA-3’  Rev 5’-CCCAAGAAACCCTAACACCA-3’ |
| TC225712 | Solyc03g114840 | For 5’-CCAACGTTGCAGCTATGCTA-3’  Rev 5’-AGATCGTTGGAGGAGCTCAA-3’ |
| TC226086 | Solyc03g095770 | For 5’-AGTTTAACCCAGGGCCAGAT-3’  Rev 5’-TCTCCATGCACAACCATCAT-3’ |
| TC226660 | Solyc06g035940 | For 5’-AGAGATGGTGGCGTATCTGG-3’  Rev 5’-TGTTGCGAGCTCTTGTTTTG-3’ |
| TC227487 | Solyc08g008280 | For 5’-AGCCAGATGAATGGTTACGG-3’  Rev 5’-ACCCACAATTGGAGAACTCG-3’ |
| TC228072 | Solyc01g102980 | For 5’-CTTGTCCCTTTTGGTCTCCA-3’  Rev 5’-TGTTGGGTGGTGTAGGATCA-3’ |
| TC229525 | Solyc07g053030 | For 5’-GGTGATCGTTCATCCATCCT-3’  Rev 5’-TCCTGGCATTAAAAGGCTGT-3’ |
| TC230482 | Solyc04g071610 | For 5’-TACCGGGAACATCCGATTAG-3’  Rev 5’-TTGGCATGAATCGAGGAAAT-3’ |
| TC230937 | Solyc01g073810 | For 5’-TACTATTGCGGTGGGTGTGA-3’  Rev 5’-GAATTCCGACTGGGATTGAA-3’ |
| TC233540 | Solyc06g075960 | For 5’-AATGTCTGGTCGTGGAAAGG-3’  Rev 5’-CCTGCGAATAGCTGGCTTAG-3’ |
| TC236211 | Solyc04g079940 | For 5’-TTTGTGGAATGATGCAGAGC-3’  Rev 5’-CCCATCGTTACCGCTAGTGT-3’ |
| TC238621 | Solyc07g052730 | For 5’-TTCCCAAGAATCCTGTCCTG-3’  Rev 5’-TTGAGCTCTCGTCGTCTGAA-3’ |
| TC239866 | Solyc05g014280 | For 5’-TGAAGGTCATGGTTGTTGATG-3’  Rev 5’-TTGGACTGTCCTTGCTGTTG-3’ |
