# Supplementary material for: Transcriptomic analysis reveals the gene regulatory networks involved in leaf and root response to osmotic stress in tomato
Source: Front Plant Sci. 2023 Jun 2;14:1155797. doi: 10.3389/fpls.2023.1155797 (PMC10272567; doi:10.3389/fpls.2023.1155797)
Supplement: Supplementary Table 8 — Log2 Fold Change of selected DETs from microarray and qRT-PCR data used for validation of gene expression data. [file Table_8.docx]

|  |  | **ARRAY** | **q-PCR** |
| --- | --- | --- | --- |
| **Sample** | **Gene ID** | **Log2 Fold Change** | **Log2 Fold Change** |
| Leaf M82 | Solyc03g116100 | 1.737 | 2.733 |
| Leaf M82 | Solyc07g056670 | -1.309 | -1.985 |
| Leaf M82 | Solyc07g052700 | 1.252 | 1.161 |
| Leaf M82 | Solyc12g010380 | -1.398 | -2.529 |
| Leaf M82 | Solyc01g102980 | -1.469 | -2.363 |
| Leaf M82 | Solyc04g071610 | 1.870 | 4.008 |
| Leaf M82 | Solyc05g014280 | 2.150 | 3.425 |
| Leaf Tondo | Solyc03g116100 | 1.775 | 1.419 |
| Leaf Tondo | Solyc07g056670 | -1.066 | -2.974 |
| Leaf Tondo | Solyc07g052700 | 1.458 | 1.044 |
| Leaf Tondo | Solyc12g010380 | -1.284 | -2.646 |
| Leaf Tondo | Solyc01g102980 | -1.172 | -1.747 |
| Leaf Tondo | Solyc04g071610 | 1.901 | 2.614 |
| Leaf Tondo | Solyc05g014280 | 1.843 | 1.565 |
| Root M82 | Solyc03g116100 | 3.059 | 3.702 |
| Root M82 | Solyc01g095030 | 1.065 | 1.545 |
| Root M82 | Solyc07g056670 | 1.361 | 2.174 |
| Root M82 | Solyc07g052700 | 1.041 | 0.805 |
| Root M82 | Solyc08g008280 | 1.240 | 1.482 |
| Root M82 | Solyc07g053030 | -1.293 | -1.108 |
| Root M82 | Solyc04g071610 | 1.494 | 1.109 |
| Root M82 | Solyc05g014280 | 4.619 | 5.980 |
| Root Tondo | Solyc03g116100 | 2.531 | 2.061 |
| Root Tondo | Solyc01g095030 | 1.137 | 1.217 |
| Root Tondo | Solyc07g052700 | 1.294 | 0.778 |
| Root Tondo | Solyc04g071610 | 1.460 | 0.766 |
| Root Tondo | Solyc01g073810 | -1.292 | -2.757 |
| Root Tondo | Solyc05g014280 | 3.768 | 4.508 |
